# Supplementary material for: Covalent Targeting Leads to the Development of a LIMK1 Isoform-Selective Inhibitor
Source: J Med Chem. 2025 Jul 2;68(14):15026–49. doi: 10.1021/acs.jmedchem.5c01204 (PMC12305648; doi:10.1021/acs.jmedchem.5c01204)
Supplement: Supplementary file 1 [file jm5c01204_si_001.pdf]

Supporting Information

Covalent targeting leads to the development of a LIMK1 isoform-selective inhibitor

Sebastian Mandel[a,b#], Thomas Hanke[a,b#], Niall Prendiville[e], María Baena-Nuevo[e], Lena Marie Berger[a,b], Frederic Farges[a,b], Martin Peter Schwalm[a,b,d], Benedict-Tilman Berger[a,b], Andreas Kraemer[a,b], Lewis Elson[a,b], Hayuningbudi Saraswati[c], Kamal R. Abdul Azeez[a,b], Verena Dederer[a,b], Sebastian Mathea[a,b], Ana Corrionero[e], Patricia Alfonso[e], Sabrina Keller[f], Matthias Gstaiger[f], Daniela S. Krause[c,d], Susanne Müller[a,b,d], Sandra Röhm[a,b*], Stefan Knapp[a,b,d*]

[a] *Institute for Pharmaceutical Chemistry, Johann Wolfgang Goethe-University, Max-von-Laue-Str. 9, D-60438 Frankfurt am Main, Germany*

[b] *Structure Genomics Consortium Buchmann Institute for Molecular Life Sciences, Johann Wolfgang Goethe-University, Max-von-Laue-Str. 15, D-60438 Frankfurt am Main, Germany*

[c] *Institute of Transfusion Medicine, Transfusion Centre, Johannes Gutenberg University Medical Center, 55131 Mainz and Research Center for Immunotherapy (FZI), University Medical Center, University of Mainz, Mainz, Germany.*

[d] *German Cancer Consortium (DKTK), German Cancer Research Center (DKFZ), DKTK site Frankfurt-Mainz, 69120 Heidelberg, Germany*

[e] *Enzymlogic, Qube Technology Park, C/Santiago Grisolía, 2, 28760, Madrid, Spain*

[f] *Institute of Molecular Systems Biology, Otto-Stern-Weg 3, 8093 Zürich, Switzerland*

**Authors for correspondence: roehm@pharmchem.uni-frankfurt.de or knapp@pharmchem.uni-franfurt.de*

#S.M. and T.H. contributed equally to this work

Table of Contents

Figure S1 Kinetic plot of LIMK1 inhibitors	Page S3
Figure S2 Correlation of in-vitro and in-cellular affinities for LIMK1	Page S3
Figure S3 LIMK affinities and binding kinetics in cells	Page S3
Figure S4 NanoBRET assays in HEK293T cells, negative control	Page S4
Table S1 Kinase selectivity screen, DSF-assay panel	Page S4
Table S2 Kinase selectivity screen, NanoBRET-K192 panel	Page S5-S7
Figure S5–S52 NMR spectra and MS data of synthesized compounds	Page S7-S31

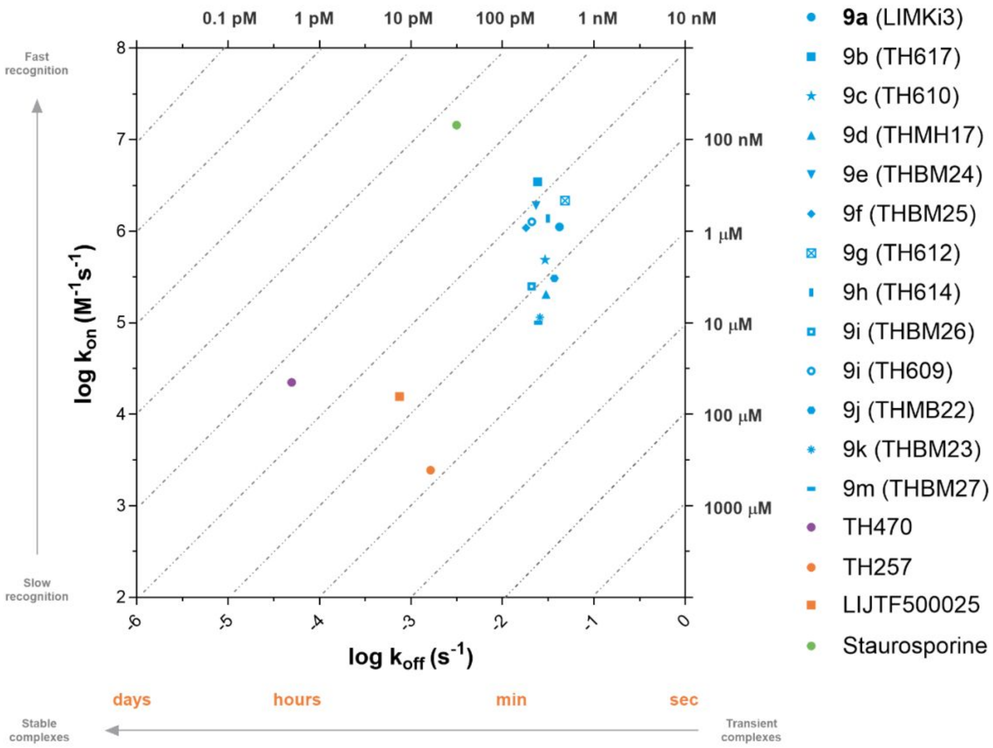

Figure S1. Kinetic plot of LIMK1 inhibitors. TR-FRET KINETICfinder assays were used to determine the kinetics for the binding of compounds to the activated form of LIMK1. The on-rate (k_{on}) is shown on the y-axis and the off-rate (k_{off}) on the x-axis, both in logarithmic scale. The dashed lines refer to the K_d values. Binding modes: blue, type-I; purple, type-II; orange, type-III. The reference compound is shown in green. In summary, type-I inhibitors, targeting the active conformation of LIMK1, had faster k_{on} and k_{off}-rates than type-II inhibitors targeting the inactive conformation. The allosteric type III inhibitor TH470 exhibited a notably slow off-rate.

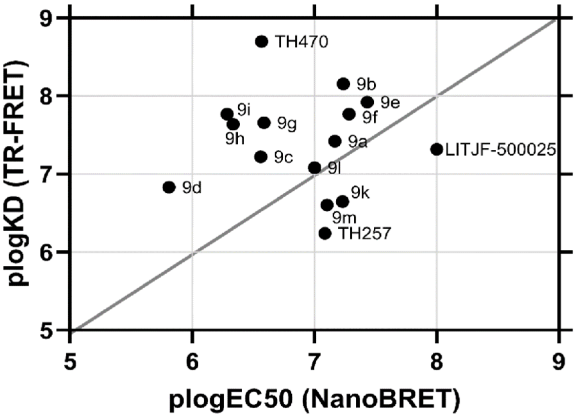

Figure S2. Correlation of in-vitro and in-cellular affinities for LIMK1. Comparison of TR-FRET KINETICfinder assays with NanoBRET assays in HEK293T cells.

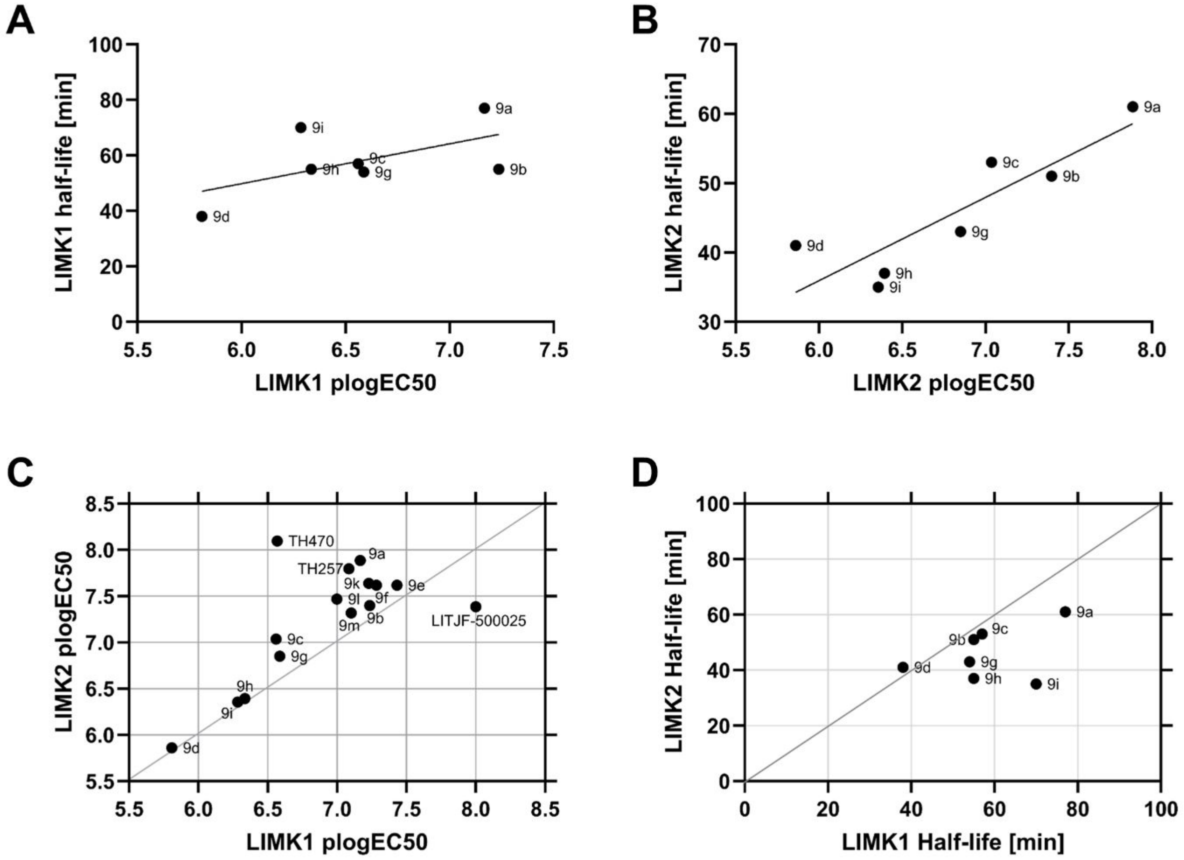

Figure S3. LIMK affinities and binding kinetics in cells. Comparison of cellular potency vs half-life for LIMK1 (A) and LIMK2 (B) derived from cellular NanoBRET assays. (C) Correlation of cellular affinities for LIMK1 vs LIMK2. (D) Correlation of the cellular half-life of LIMK1 vs LIMK2.

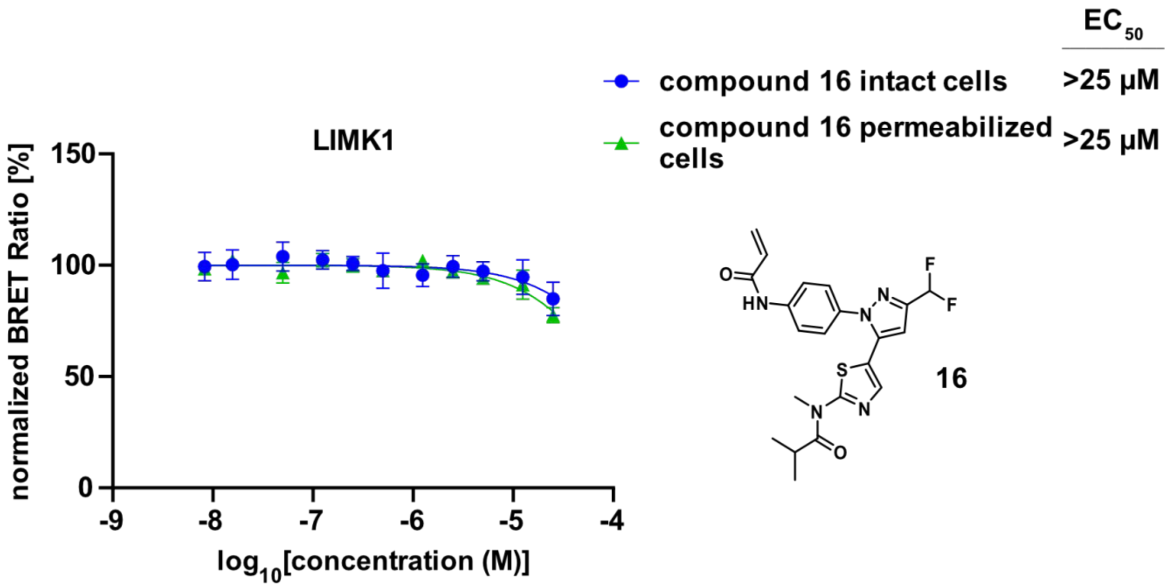

16

Figure S4. NanoBRET assays in HEK293T cells. Dose-response assays for negative control **16** (SM576, n=3).

Table S1. DSF-assay screen of compound **10** against 91 kinases at 20 µM. ΔT_m values are depicted in kelvin [K].

Target	10, ΔT_m [K]	Staurosporine (pos. control)	Target	10, ΔT_m [K]	Staurosporine pos. control
JNK3	8.12	6.61	CDKL1	0.48	5.66
LIMK1	8.11	n.d.	DCAMKL1	0.48	13.08
JNK1	7.36	6.53	STK39	0.48	7.80
BMX	2.91	7.81	BMPR2	0.46	2.71
EPHA7	2.87	13.26	DMPK1	0.44	10.16
NEK7	2.41	1.22	PLK4	0.44	18.91
CAMK2D	2.28	19.28	MAPK1	0.43	2.14
MAPK14	2.15	3.22	EPHA4	0.41	5.98
AAK1	2.01	13.12	FES	0.37	8.31
STK17B	1.83	12.92	GPRK5	0.35	7.72
TTK	1.81	11.58	TLK1	0.32	9.95
STK6	1.62	20.11	EPHA2	0.3	7.01
GAK	1.48	9.41	CHEK2	0.29	18.16
PAK4	1.48	14.88	CLK3	0.28	3.74
OSR1	1.41	7.68	DAPK3	0.28	16.56
EPHA5	1.38	8.36	BMP2K	0.27	18.42
MAP3K5	1.35	17.86	RPS6KA5	0.26	13.05
MARK4	1.35	17.74	CSNK2A2	0.25	4.71
CDK2	1.31	14.22	VRK1	0.24	3.09
PIM3	1.19	20.38	RPS6KA1	0.21	3.53
CAMK1D	1.17	11.43	EPHB1	0.18	5.45
GSK3B	1.08	11.25	MAP2K4	0.17	11.01
AURKB	1.07	15.19	MAP2K6	0.17	11.91

EPHB3	1.06	7.34	CDC42BPA	0.15	2.32
FGFR2	1.05	8.38	PKMYT1	0.13	0.35
LIMK2	1.02	n.d.	STK3	0.13	16.84
BRAF	0.98	0.05	ABL1	0.12	6.75
MELK	0.96	13.91	DAPK1	0.1	9.45
SRC	0.91	6.42	CAMKK2	0.09	20.76
MAPK9	0.89	3.07	GSG2	0.09	8.69
STK10	0.89	25.83	MAPKAPK2	0.09	3.41
MAP2K7	0.82	6.14	ULK3	0.03	17.37
CAMK4	0.81	7.87	HIPK2	-0.02	3.47
SLK	0.69	16.05	MSSK1	-0.03	3.51
MAP2K1	0.68	6.94	NEK1	-0.04	0.14
DYRK2	0.67	6.27	NEK2	-0.04	2.38
CLK1	0.67	11.88	PIM1	-0.05	21.41
PAK1	0.67	7.17	STK17A	-0.06	13.56
FGFR1	0.61	6.26	MAPK13	-0.08	5.84
MST4	0.60	6.51	CSNK1D	-0.09	1.74
MAPK15	0.56	15.77	CAMK2B	-0.09	14.31
PCTK1	0.55	8.88	MARK3	-0.09	18.82
CSNK1E	0.54	3.03	SRPK1	-0.15	6.12
ULK1	0.52	13.23	CASK	-0.37	3.65
DYRK1	0.52	10.29	STK4	-0.73	16.05
			MST3	-2.61	2.42

Table S2. NanoBRET-K192 panel. Compound **10** was tested at 1 µM against 192 kinases in HEK293T cells.

**Target**	**10, [1 µM] % of occupancy**	**Target**	**10, [1 µM] % of occupancy**	**Target**	**10, [1 µM] % of occupancy**
LIMK1	91.4	MARK2	19.4	BRAF(V600E)	7.6
CDKL2	84.9	ABL2	19.3	STK35	7.4
TXK	73.4	MAP3K21	18.6	AXL	7.3
NLK	73.1	IKBKE	18.1	PLK2	6.9
MAP3K19	71.1	AURKB	18.0	SGK1	5.9
AAK1	66.0	STK4	17.4	NEK2	5.8
MAPK9	64.9	CDK4	17.4	PRKCE	5.5
BMP2K	59.6	ICK	17.1	DYRK1B	5.1
STK36	49.9	BRSK2	16.7	MAST4	4.9
BMX	49.8	RIOK2	16.7	CSNK1G2	4.5
CDK3	47.3	BRSK1	16.6	STK38	4.5
LIMK2	46.9	CSNK1D	16.3	CDK10	4.1
CDKL5	46.7	AURKA	16.2	CSNK1A1L	4.0
CDKL3	46.3	MYLK4	16.1	MAPK4	3.8
CDK2	43.3	SBK3	15.9	PRKAA1	3.4

MAPK8	43.2	PHKG1	15.9	BTK	3.2
EPHA4	41.9	TIE1	15.8	TNK2(iso1)	2.5
FGFR2	41.4	CLK1	15.5	STK38L	2.5
MAP4K5	40.5	MARK4	15.4	DYRK1A	2.3
CLK4	39.4	CDK6	15.3	PRKACB	2.0
CDK1	36.5	MAP3K4	15.0	MAP4K1	1.6
EPHB1	36.4	CLK2	14.8	TBK1	1.5
CDK16	36.4	CDK18	14.8	RPS6KA3	1.4
CDK17	34.7	NEK11	14.7	FER	1.3
FGFR1	34.0	FGFR4	14.6	NEK1	1.0
EPHB4	31.8	FLT3	14.5	SIK1	0.7
NTRK2	31.5	MAP3K3	14.0	MYLK2	0.6
JAK2 (V617F)	30.8	MUSK	13.9	PRKAA2	0.4
SRMS	30.1	EPHA6	13.8	IGF1R	0.2
FYN	29.6	MLTK	13.3	RPS6KA2	0.1
NEK5	29.5	PTK2B	13.1	MAPK11	0.1
PHKG2	29.2	ERN2	13.1	IRAK3	-0.2
NEK9	28.9	PLK3	13.0	TYRO3	-0.5
STK10	28.9	CAMK2A	13.0	CSNK2A2	-1.4
FES	28.7	TEC	12.7	LTK	-2.3
DAPK2	28.5	NIM1K	12.5	MYLK3	-2.5
CDKL1	28.4	CDK7	12.4	MELK	-2.5
STK32B	27.4	PRKACA	12.4	PLK4	-2.8
TEK	27.3	PAK4	12.1	TNNI3K	-2.8
STK16	27.3	SNRK	12.1	WEE1	-3.0
CDK9	27.1	AKT2	12.0	MAP3K11	-3.5
GAK	27.0	AURKC	11.9	WEE2	-3.6
MAST3	27.0	TLK2	11.6	MAP3K10	-3.7
LCK	26.5	CAMK1	11.5	RON	-3.9
JNK3	26.4	TLK1	11.4	LATS1	-4.1
PAK6	26.2	MAP3K9	11.2	CSNK2A1	-4.4
ULK3	26.1	PTK2	11.2	RPS6KA4	-4.7
RIPK2	26.0	HIPK3	11.1	MET	-5.0
HIPK4	25.6	NUAK1	10.6	TNK1	-5.3
DCLK3	25.3	RIPK1	10.5	INSR	-5.6
MAP3K2	25.2	STK3	10.4	HIPK2	-5.8
MKNK2	24.2	NEK3	10.3	MAPK6	-6.3
CDK20	24.0	JAK3	10.2	MERTK	-6.3
NTRK1	23.9	MAP3K12	10.2	MAP4K3	-6.7
CDK5	23.4	ULK2	10.0	PRKX	-7.1
EPHA7	22.2	ERN1	9.9	ULK1	-7.5
CAMK2D	22.2	CDK15	9.5	STK33	-8.6
LRRK2	21.4	STK11	9.4	NEK4	-8.7
SIK3 FL	21.4	CDK14	9.2	MAP4K2	-13.0
SLK	20.6	FGFR3	9.1	LATS2	-17.0
EPHA1	19.9	IRAK4	9.0	RPS6KA1	-20.4

MAPK14	19.8	RET	7.8	PKMYT1	-20.9
STK17B	19.6	SNF1LK2	7.7	RPS6KA6	-33.9
ITK	19.4	PTK6	7.7	CHEK2	-194.1

NMR spectra and MS data of synthesized compounds

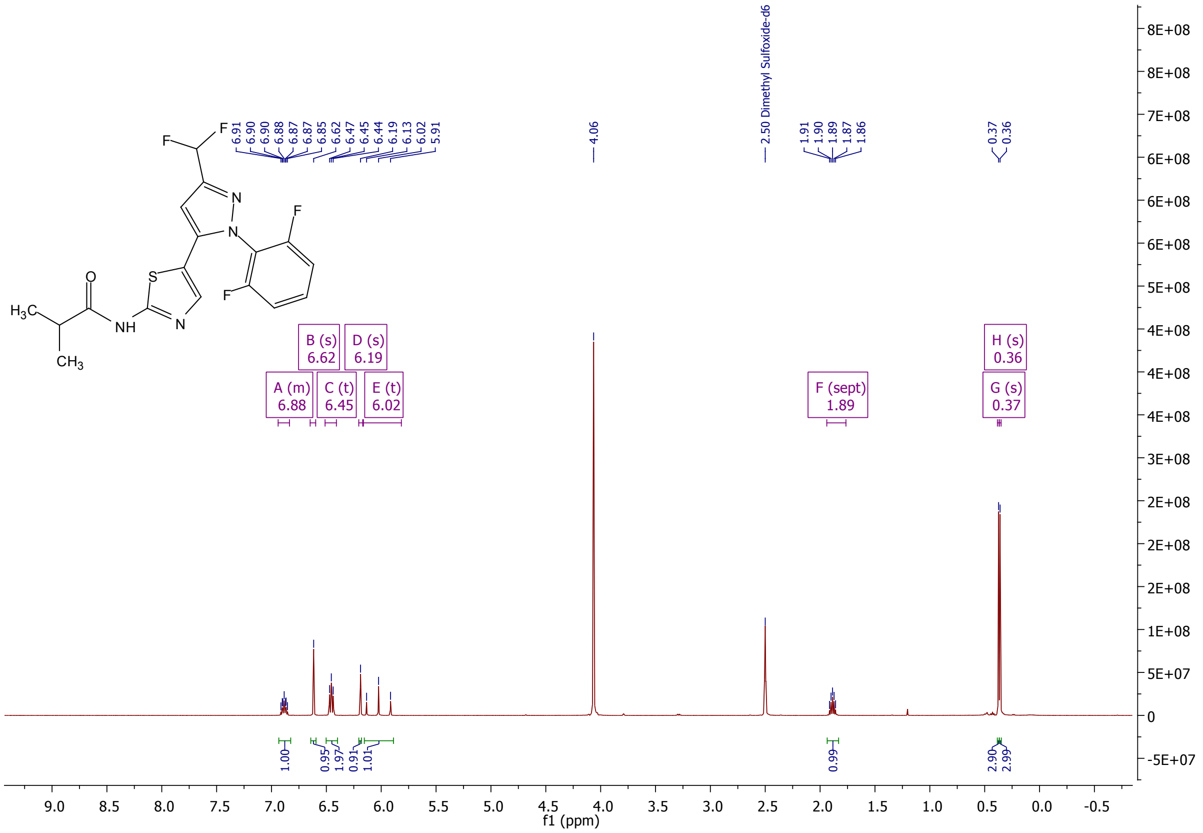

Figure S5. ^{1}H-NMR spectrum of compound 9b

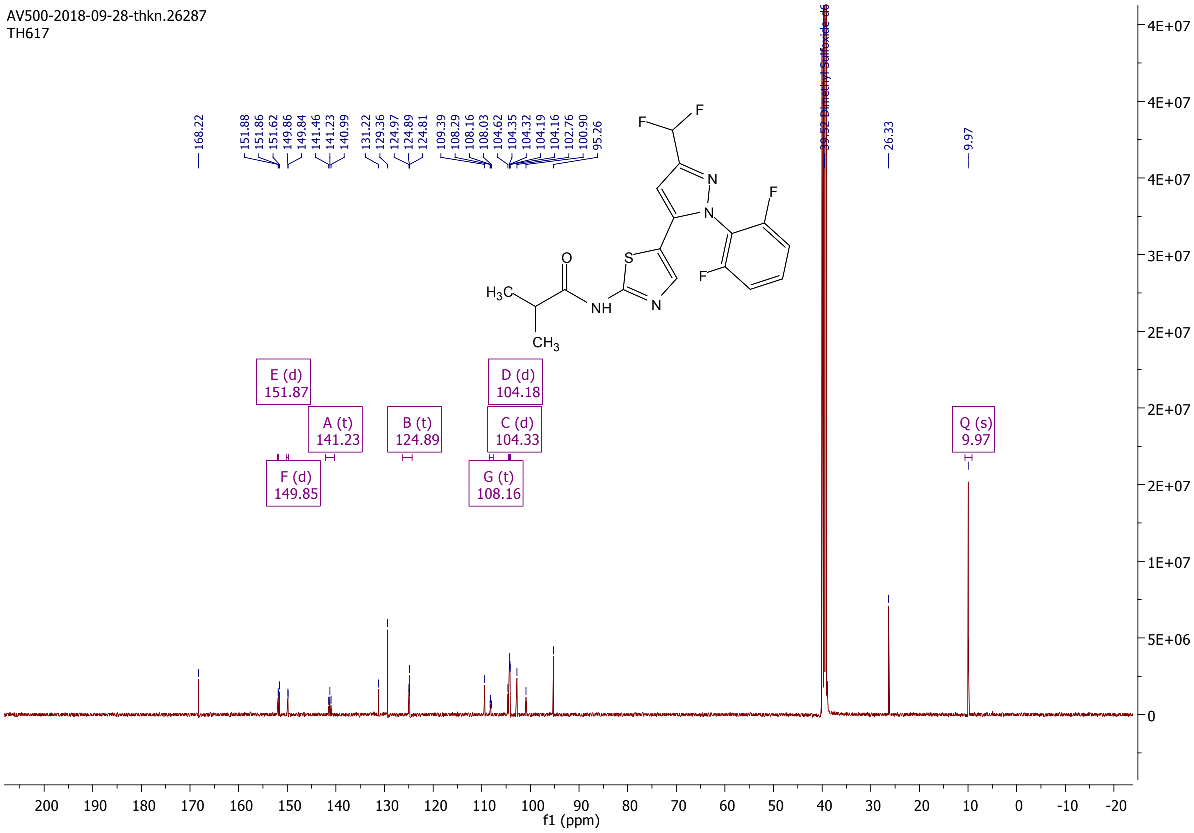

Figure S6. ^{13}C-NMR spectrum of compound 9b

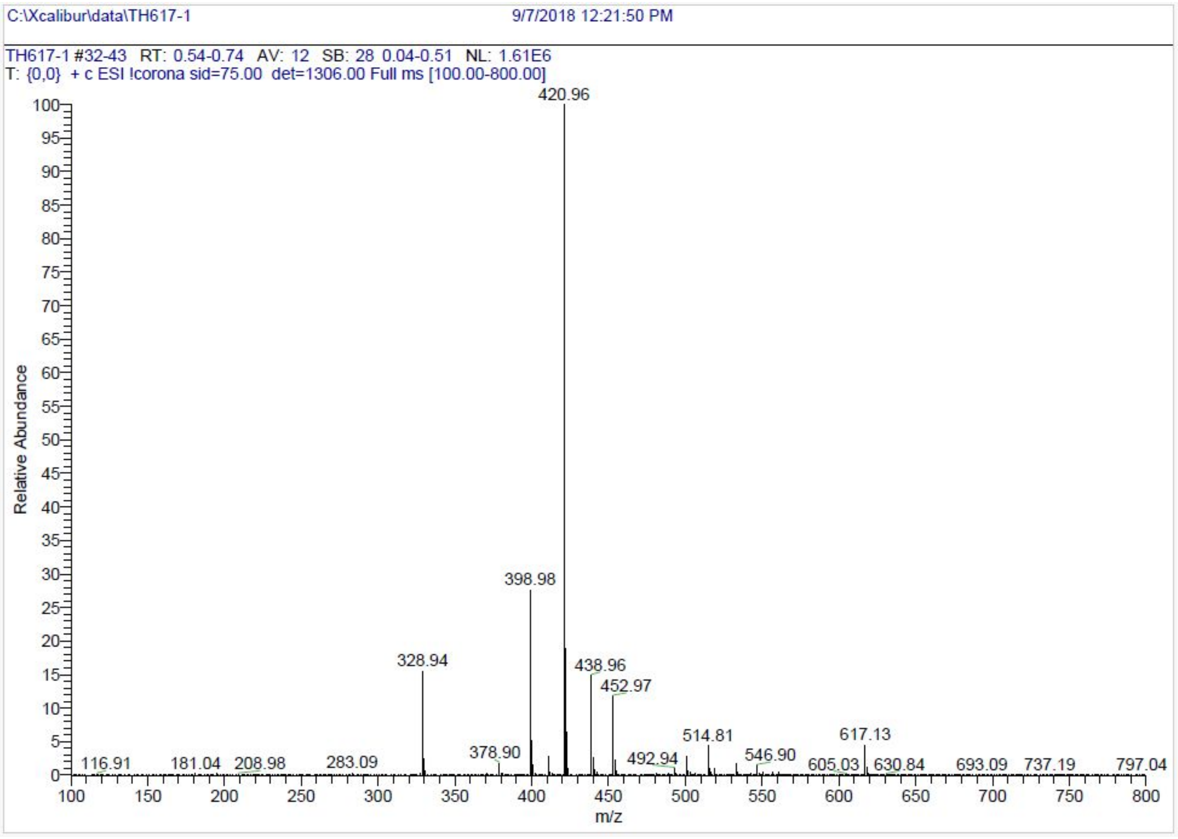

Figure S7. ESI spectrum of compound 9b

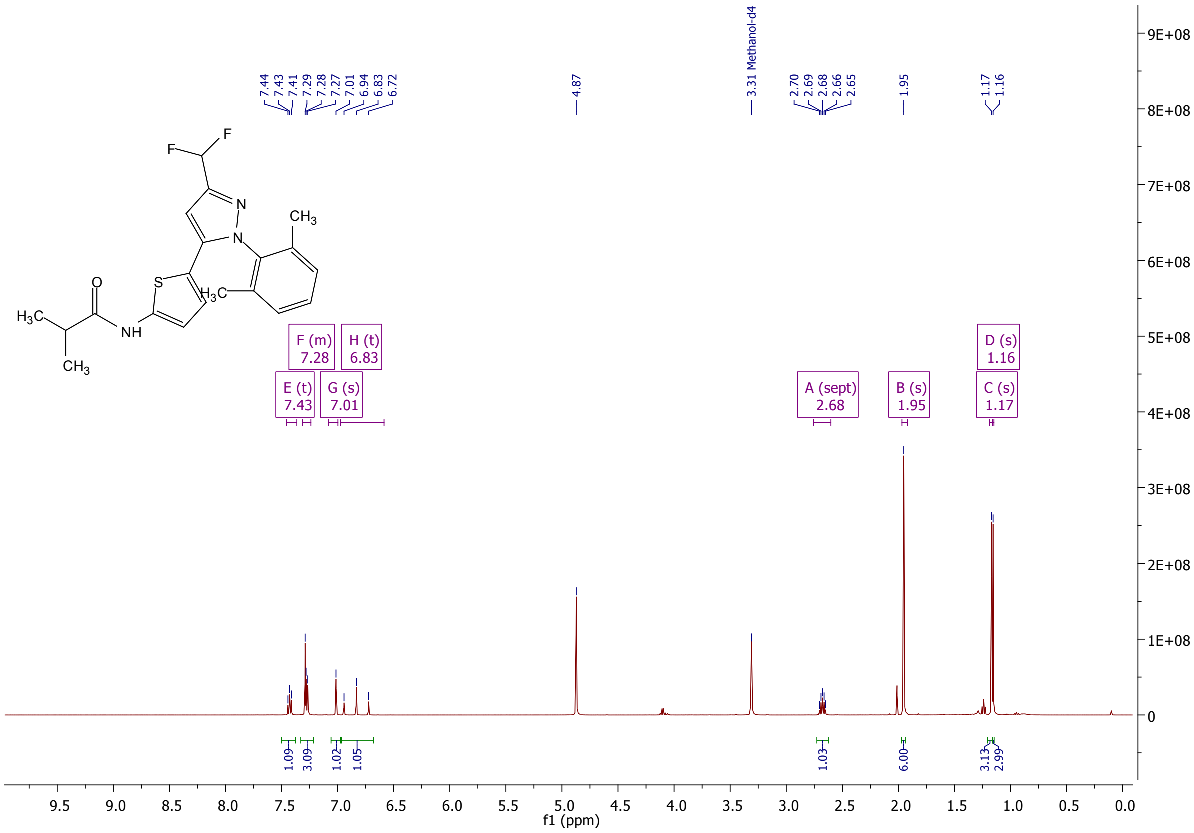

Figure S8. **^{1}H-NMR spectrum of compound 9c**

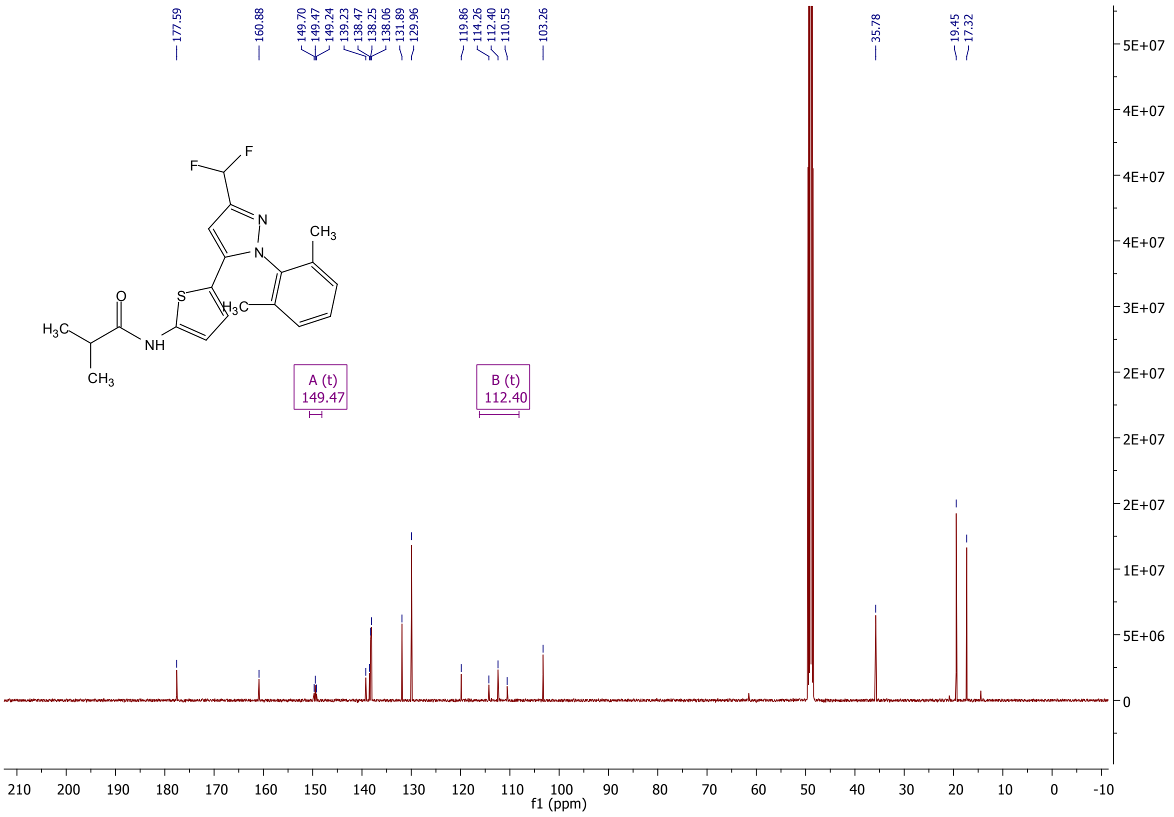

Figure S9. **^{13}C-NMR spectrum of compound 9c**

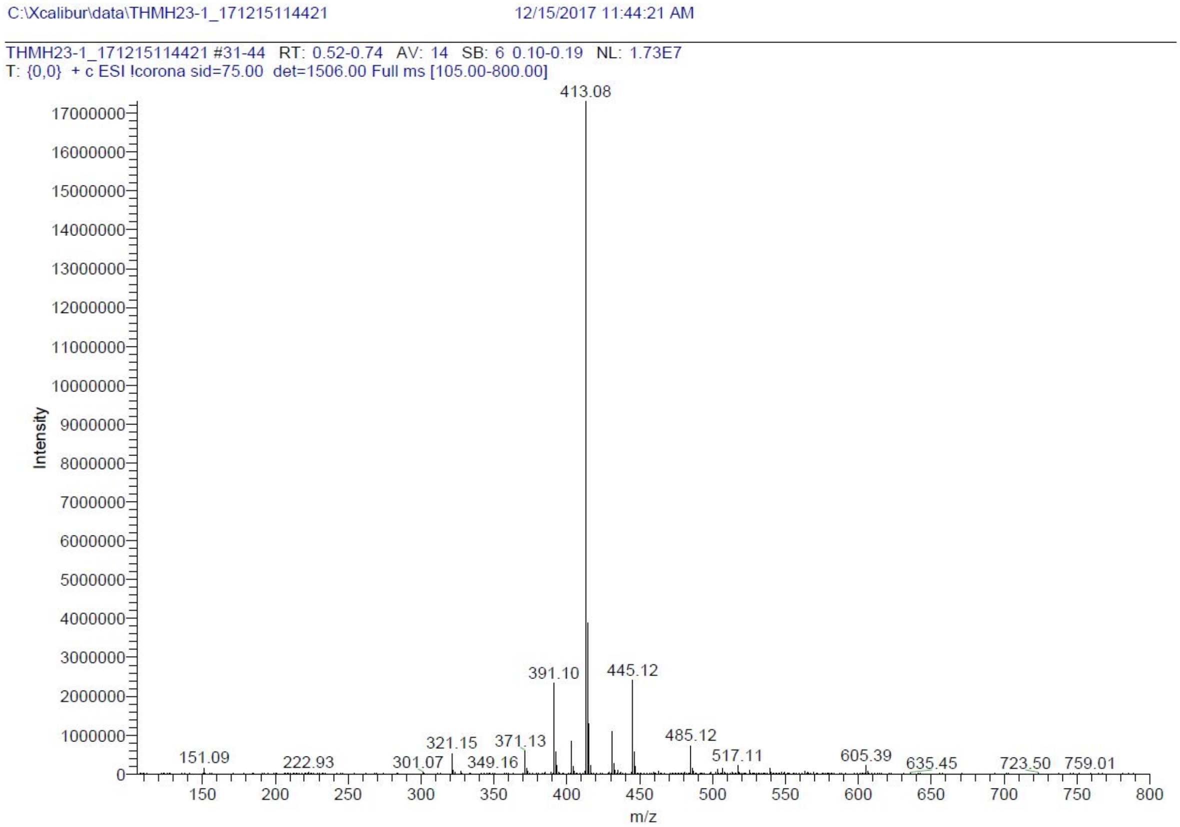

Figure S10. ESI-spectrum of compound 9c

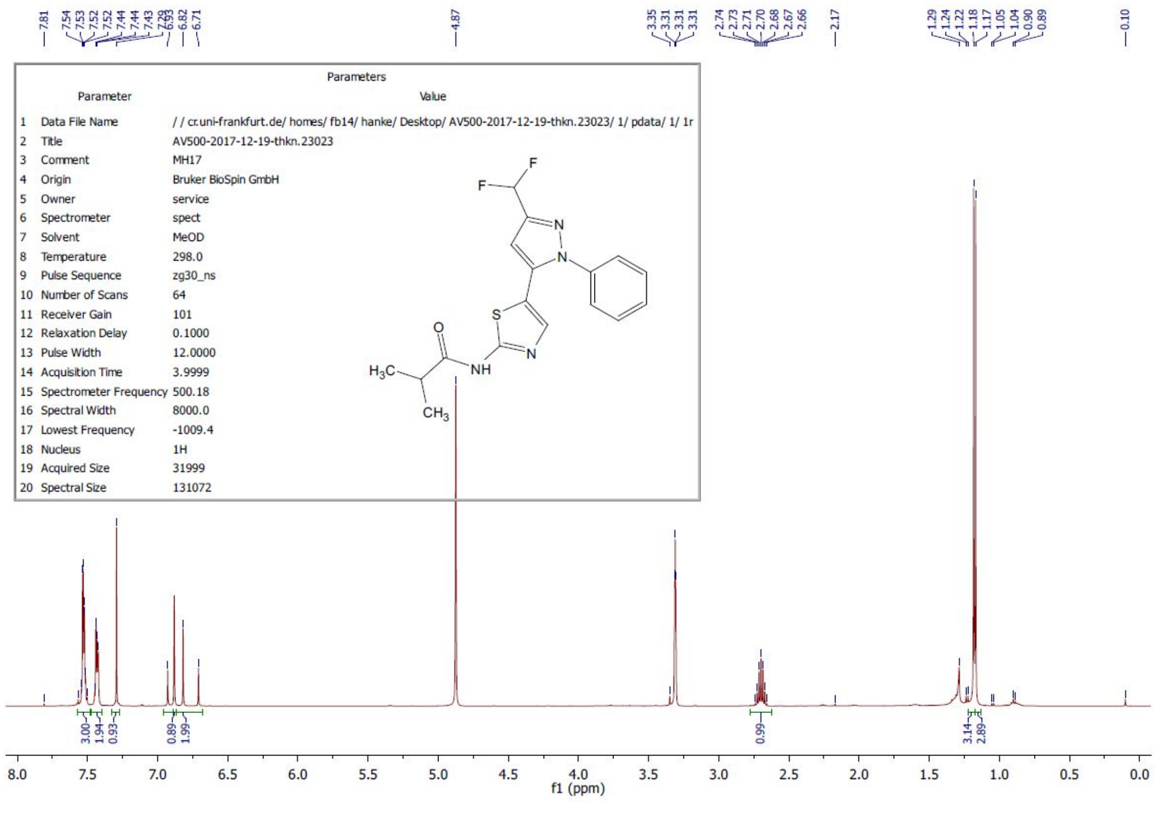

Figure S11. ^{1}H-NMR spectrum of compound 9d

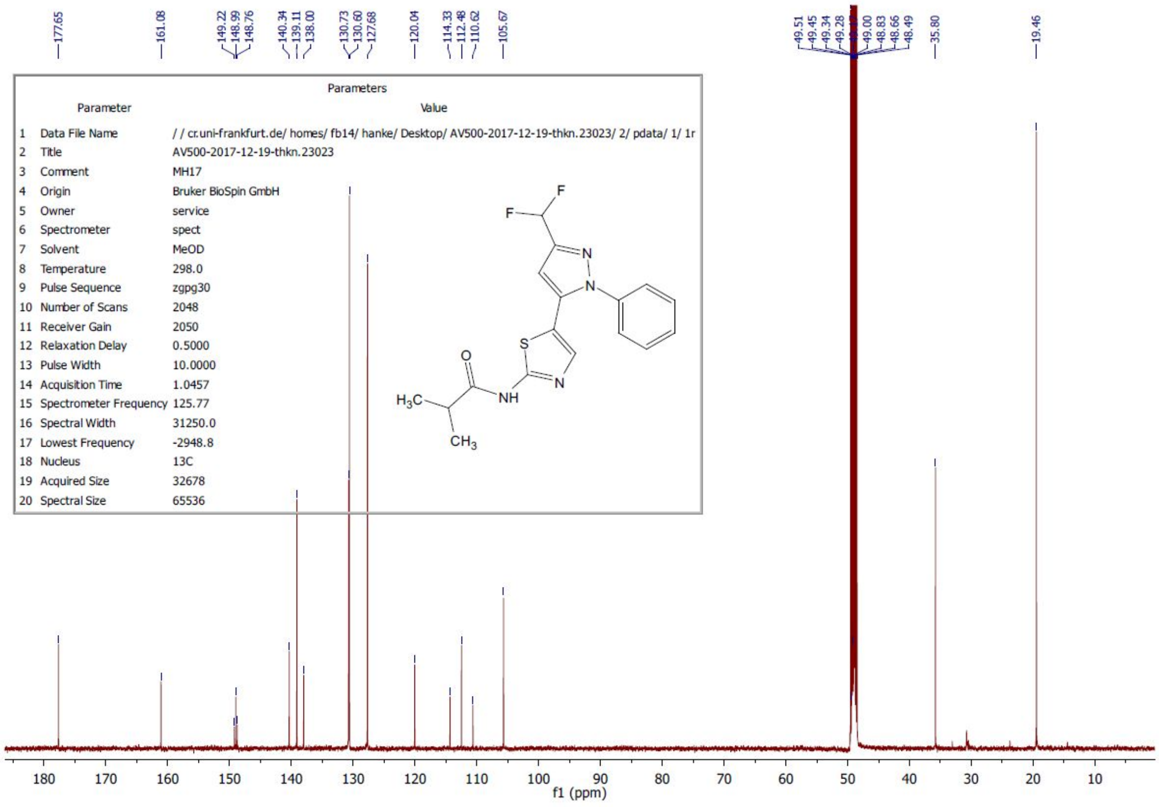

Figure S12. ^{13}C-NMR spectrum of compound 9d

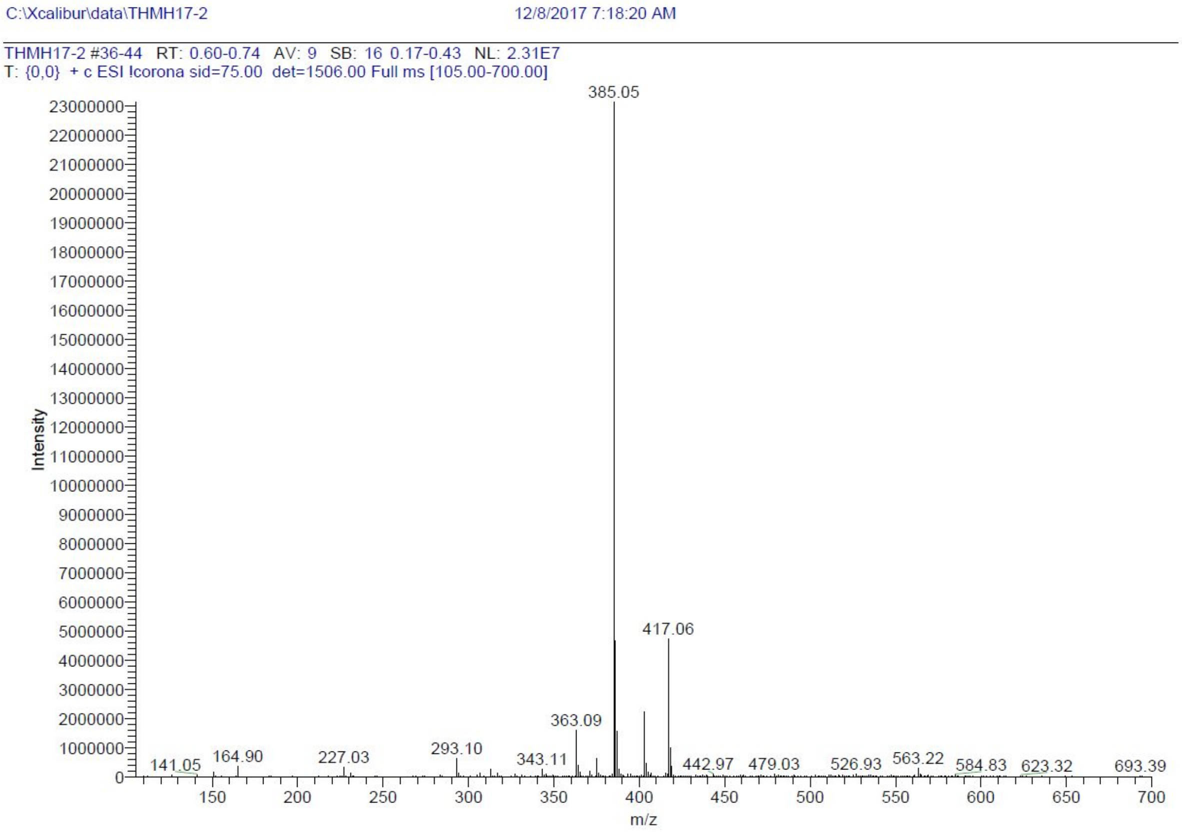

Figure S13. ESI-spectrum of compound 9d

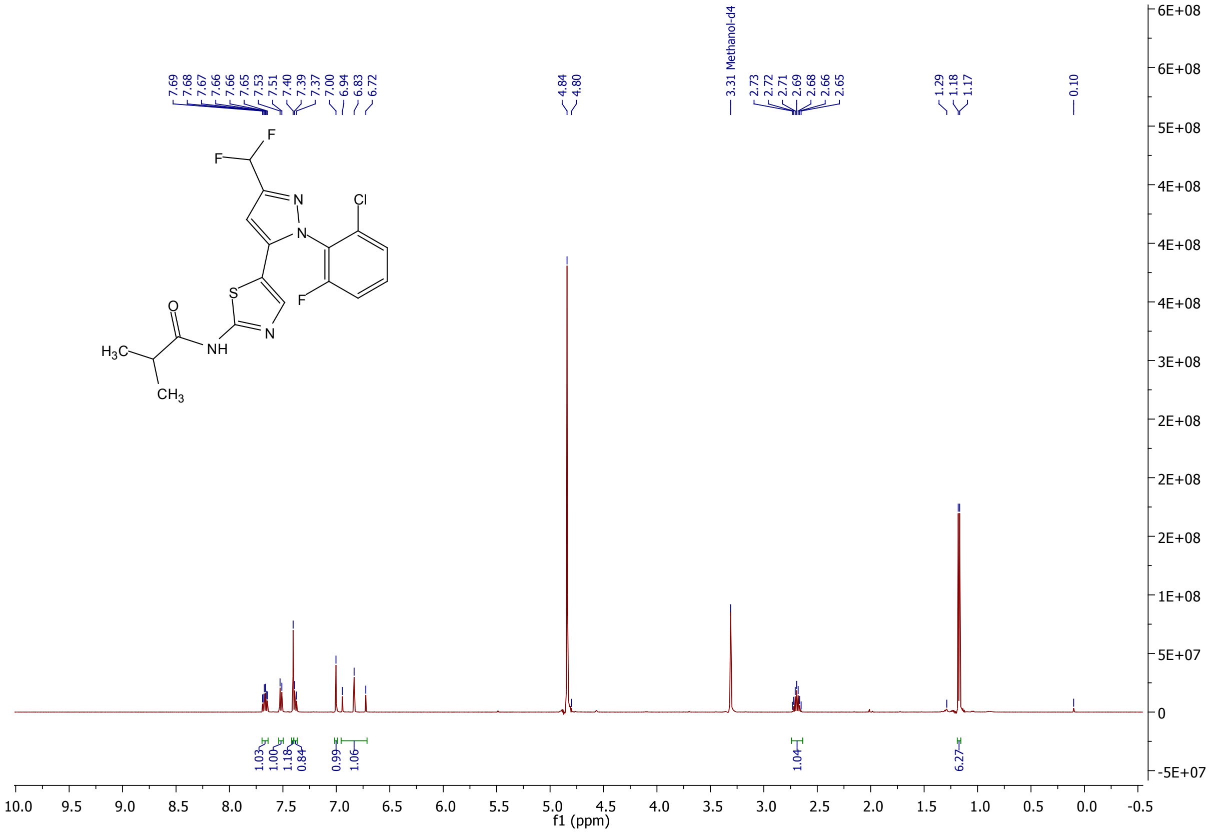

Figure S14. ^{1}H-NMR spectrum of compound 9e

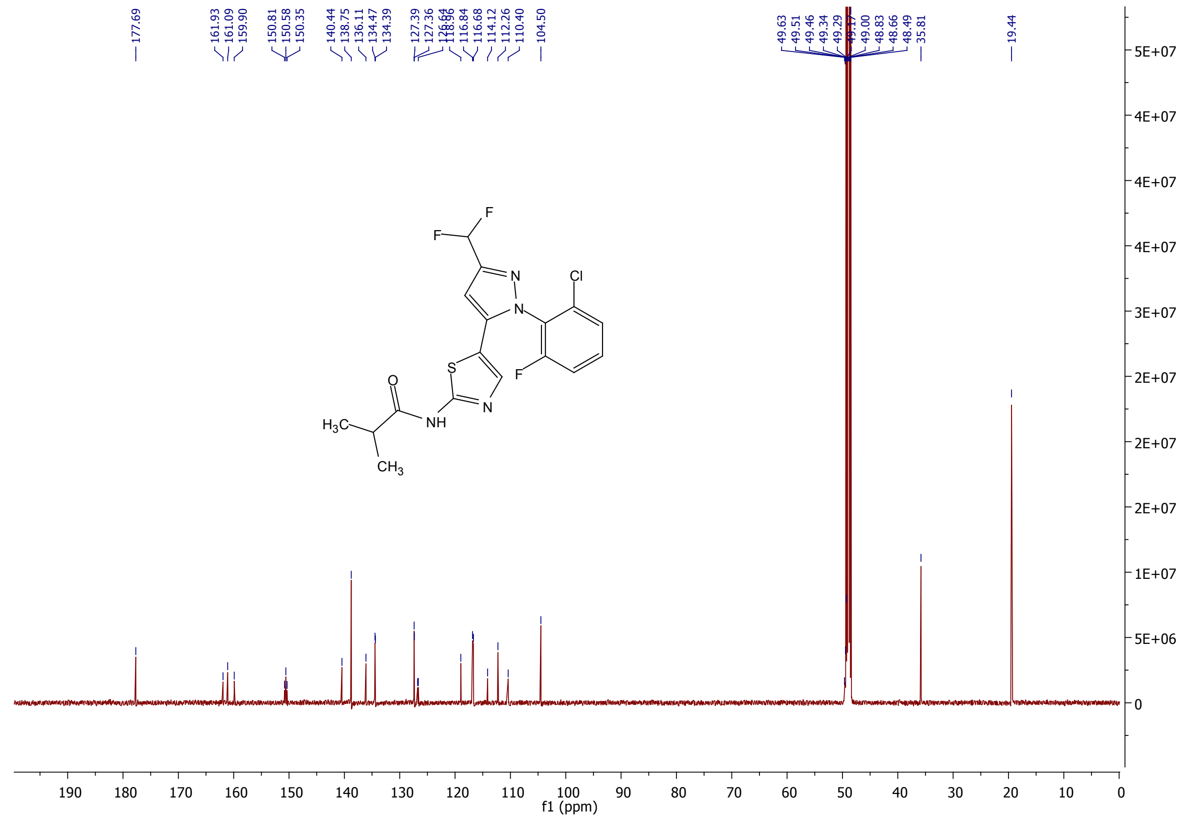

Figure S15. ^{13}C-NMR spectrum of compound 9e

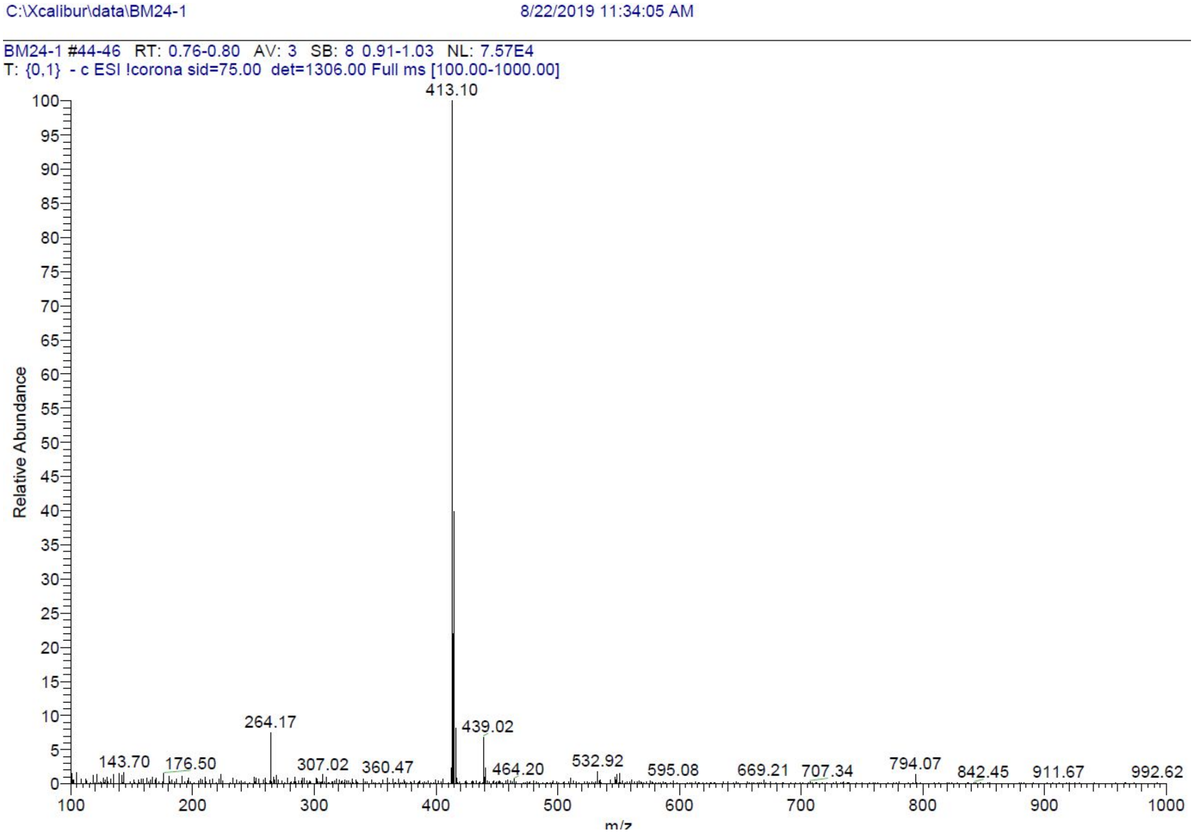

Figure S16. ESI-spectrum of compound 9e

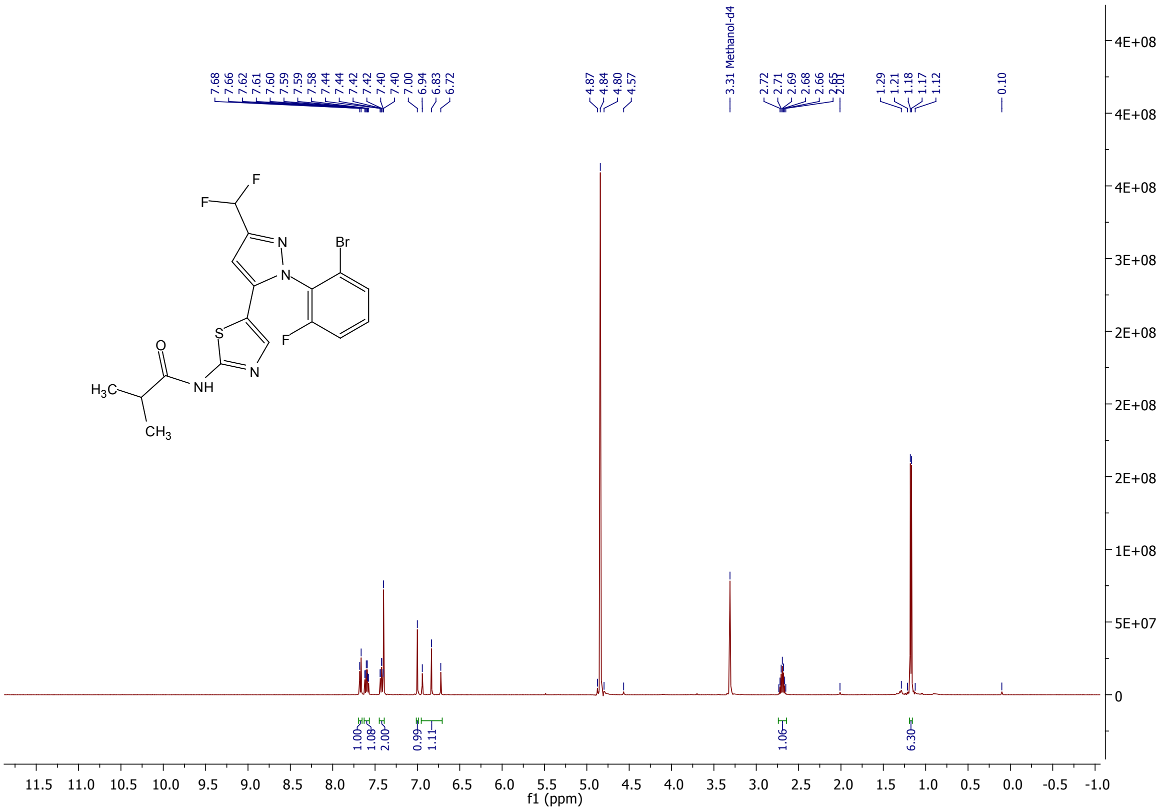

Figure S17. ^{1}H-NMR spectrum of compound 9f

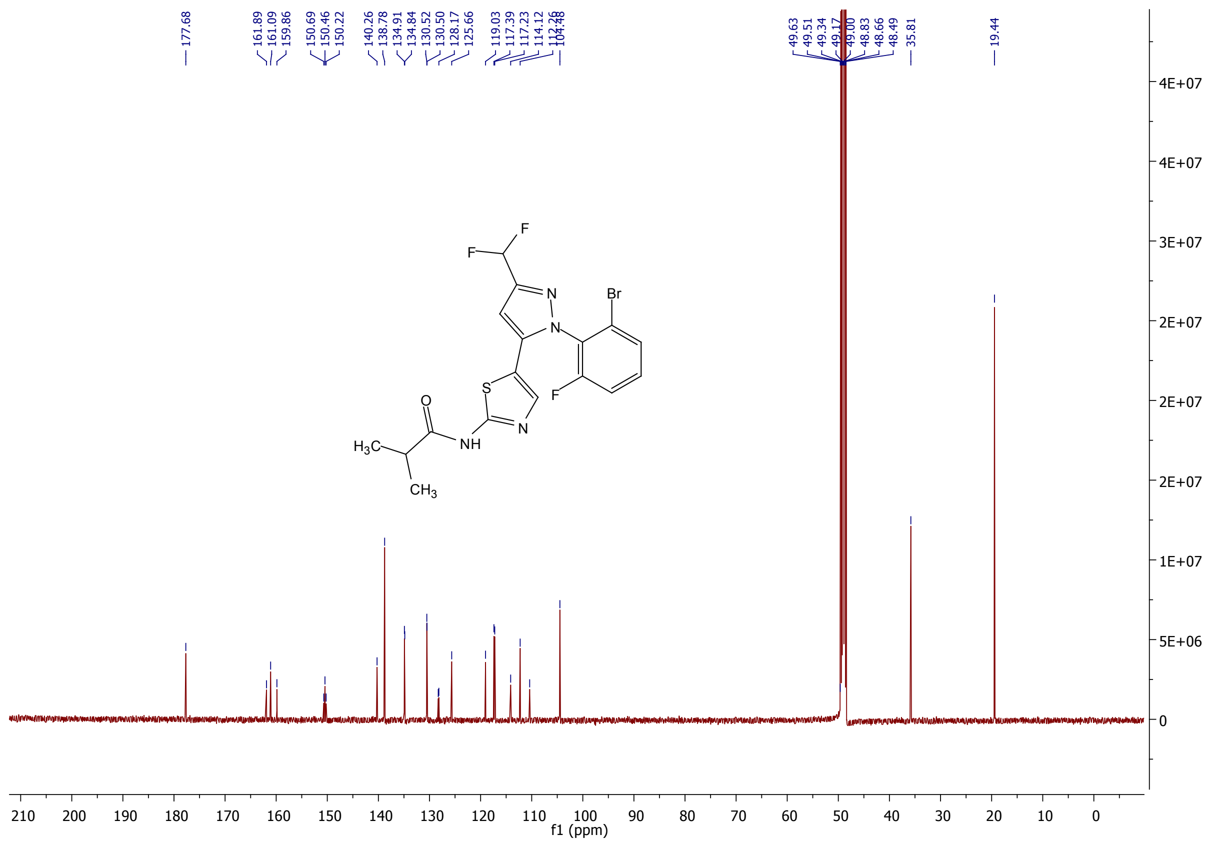

Figure S18. ^{13}C-NMR spectrum of compound 9f

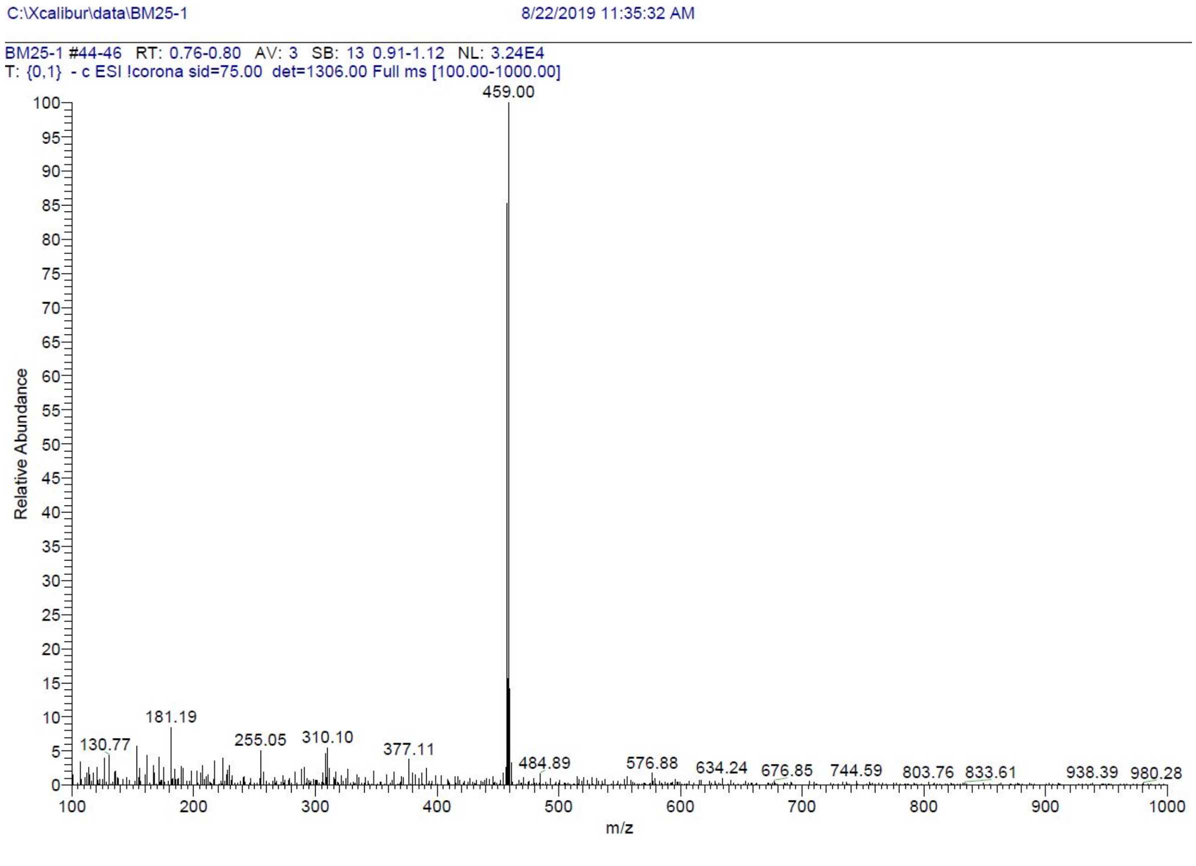

Figure S19. ESI-spectrum of compound 9f

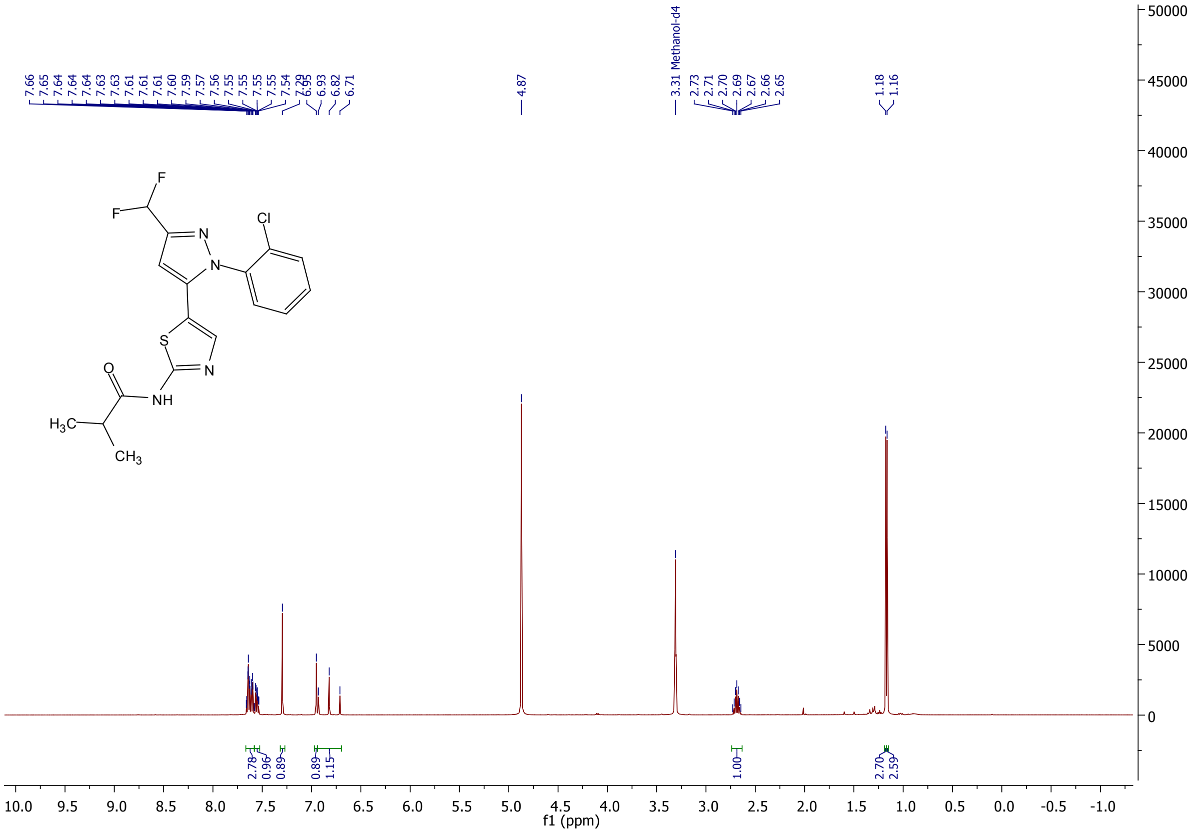

Figure S20. ^{1}H-NMR spectrum of compound 9g

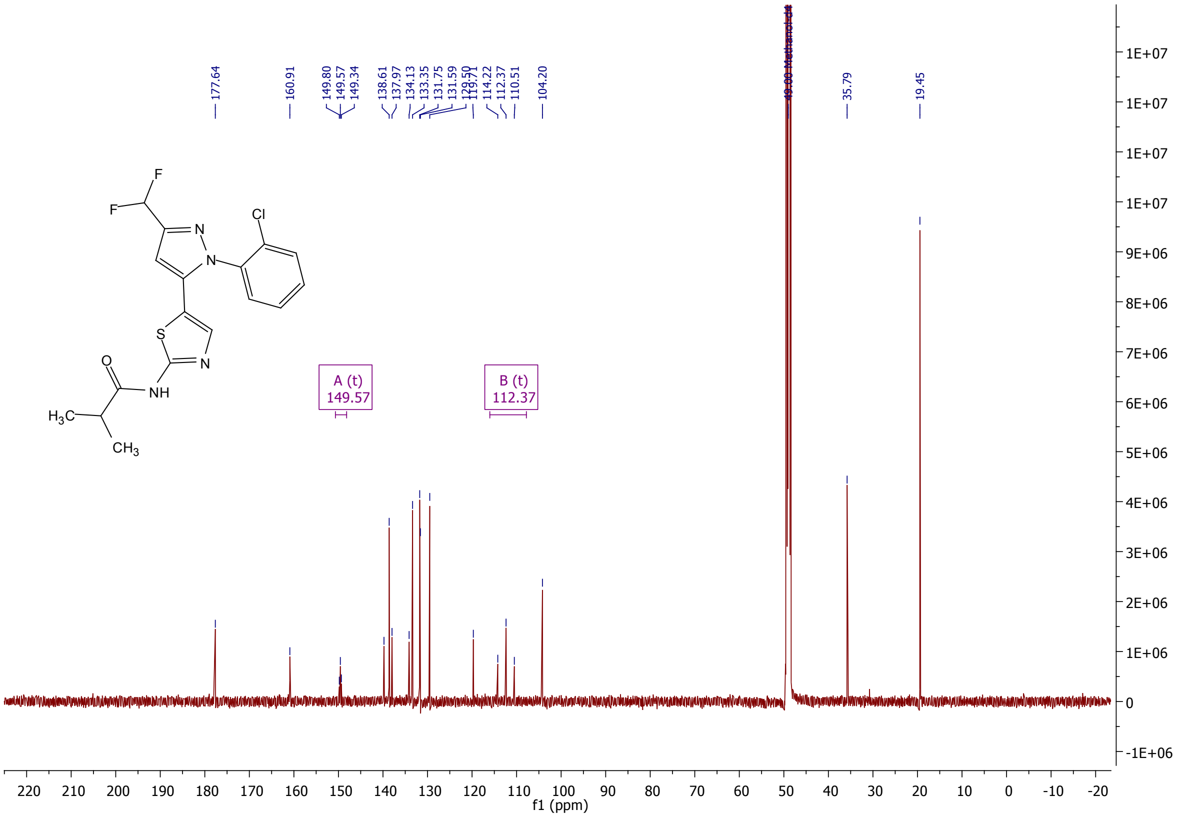

Figure S21. ^{13}C-NMR spectrum of compound 9g

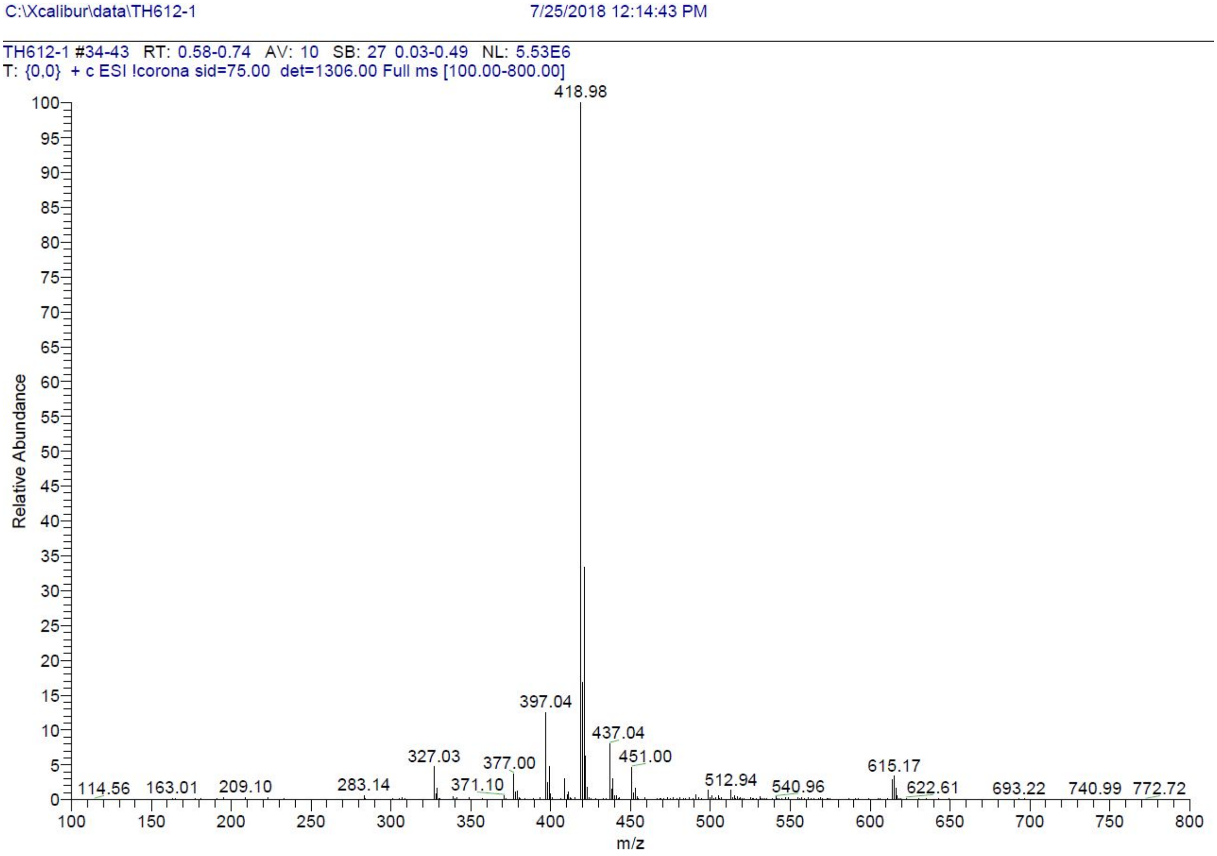

Figure S22. ESI spectrum of compound 9g

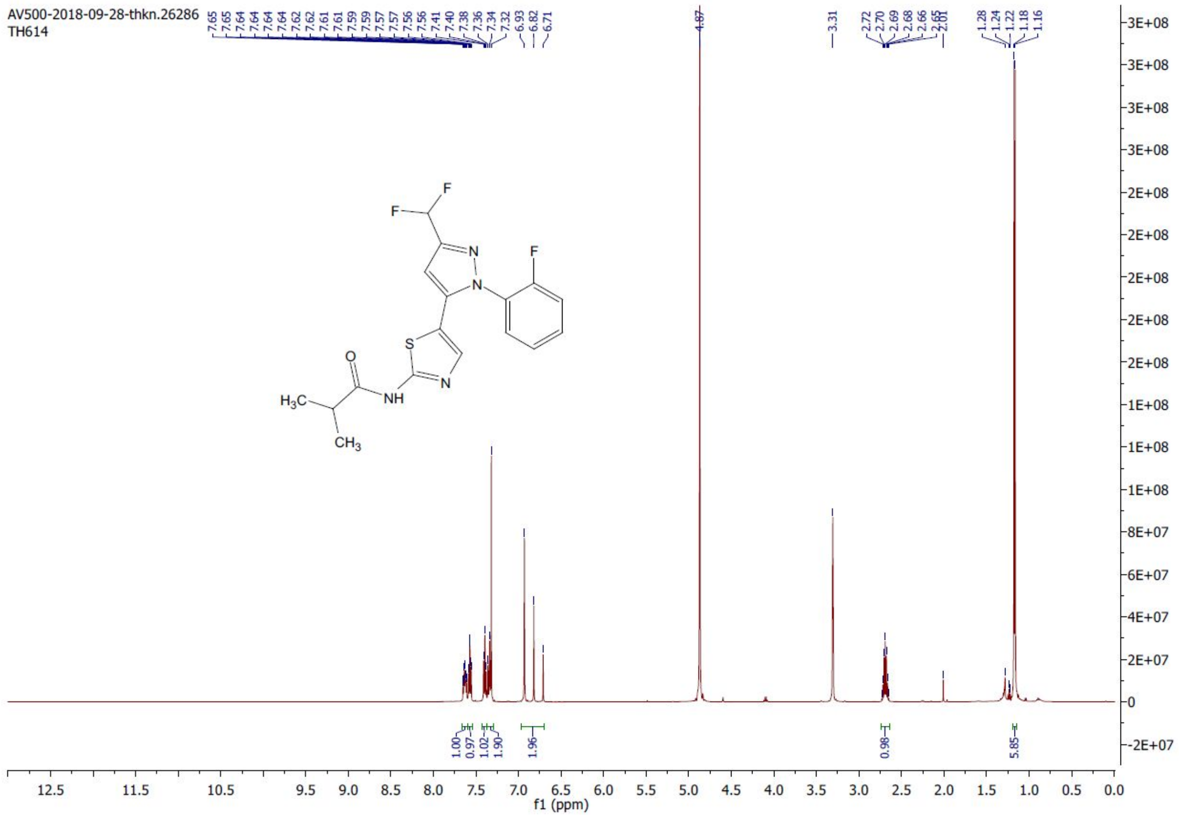

Figure S23. ^{1}H-NMR spectrum of compound 9h

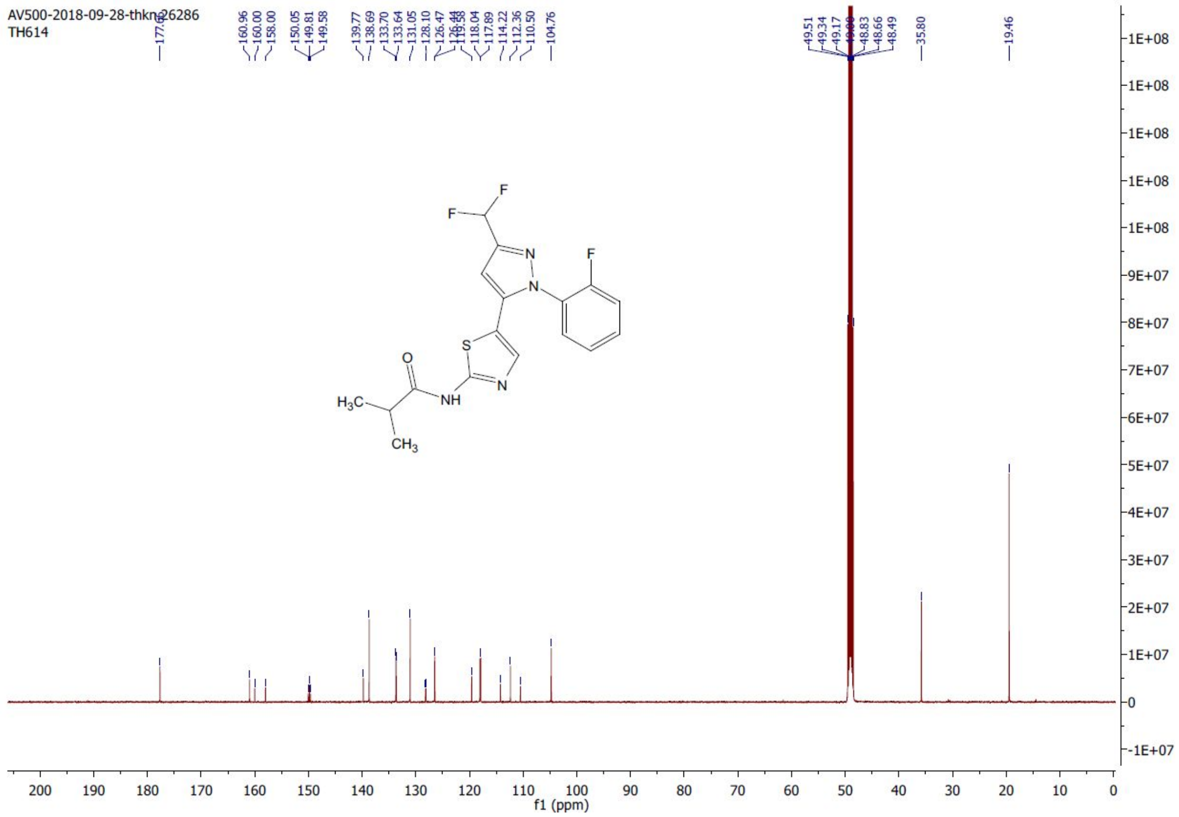

Figure S24. ^{13}C-NMR spectrum of compound 9h

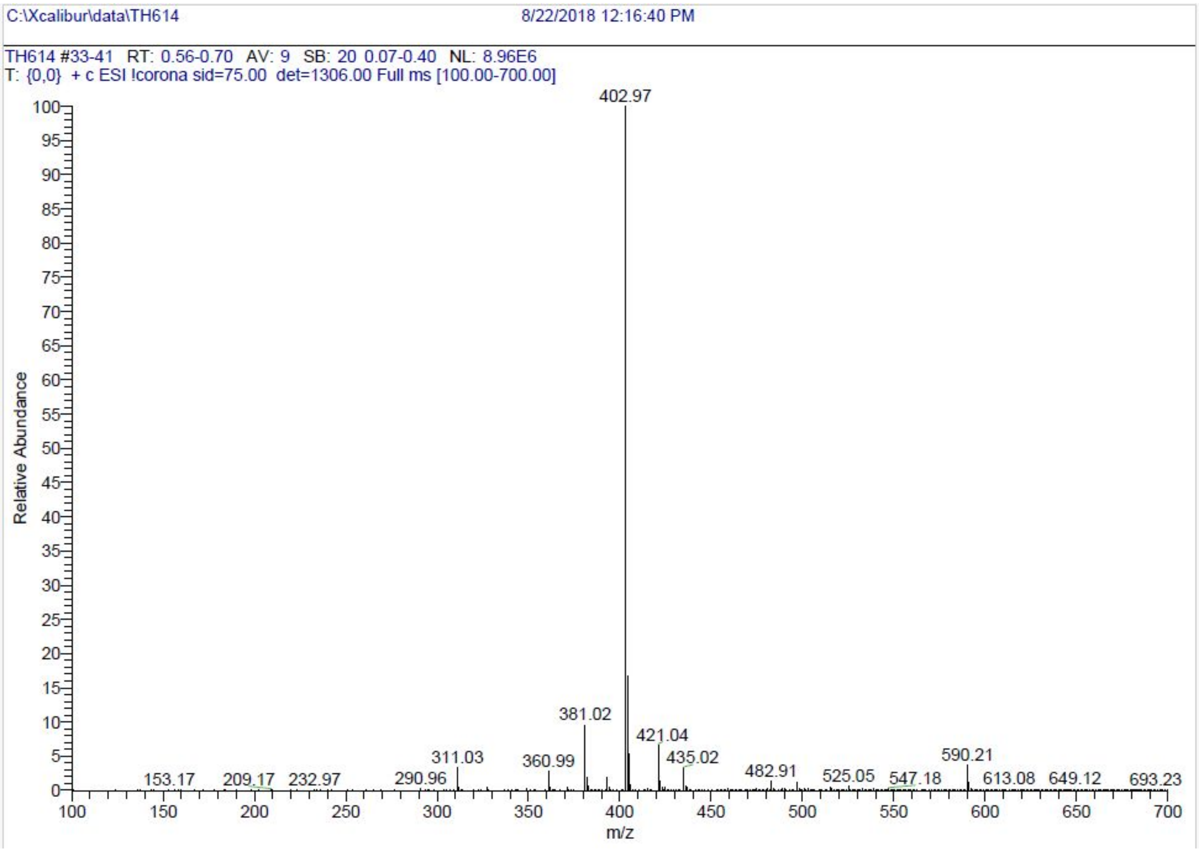

Figure S25. ESI spectrum of compound 9h

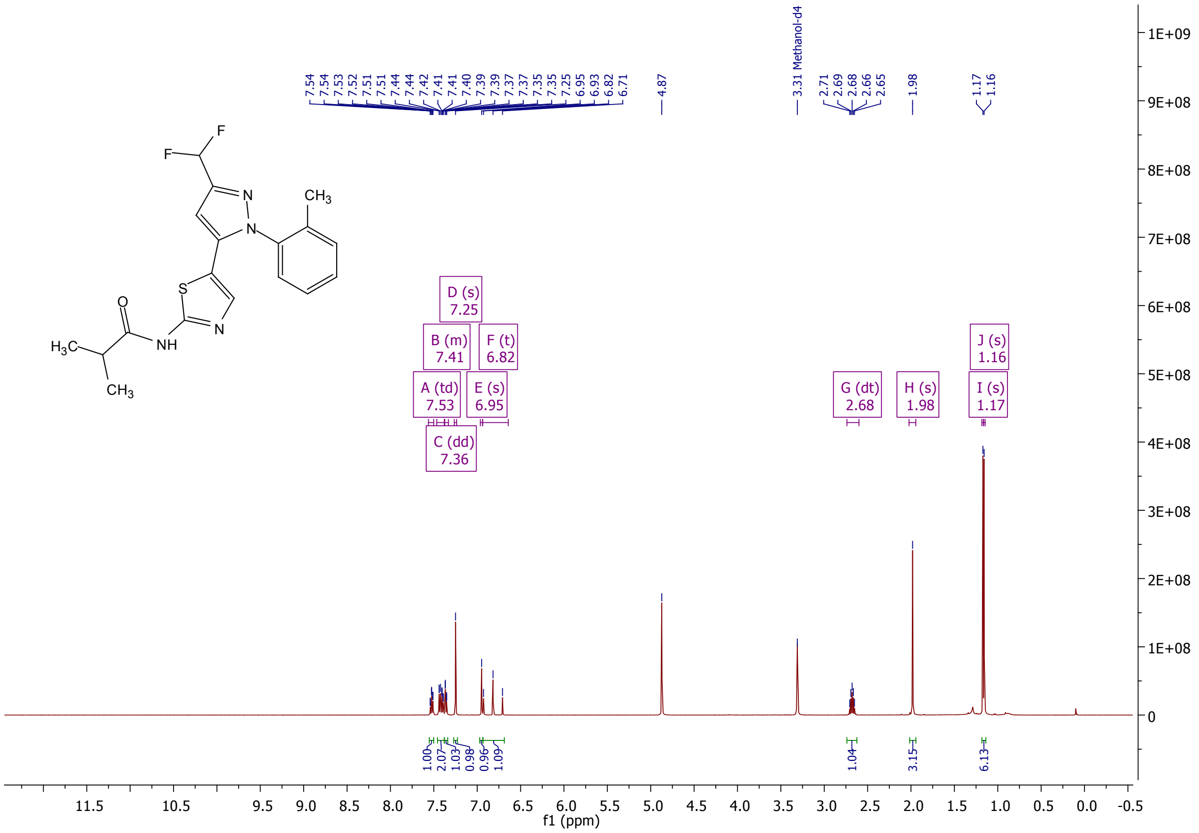

Figure S26. ^{1}H-NMR spectrum of compound 9i

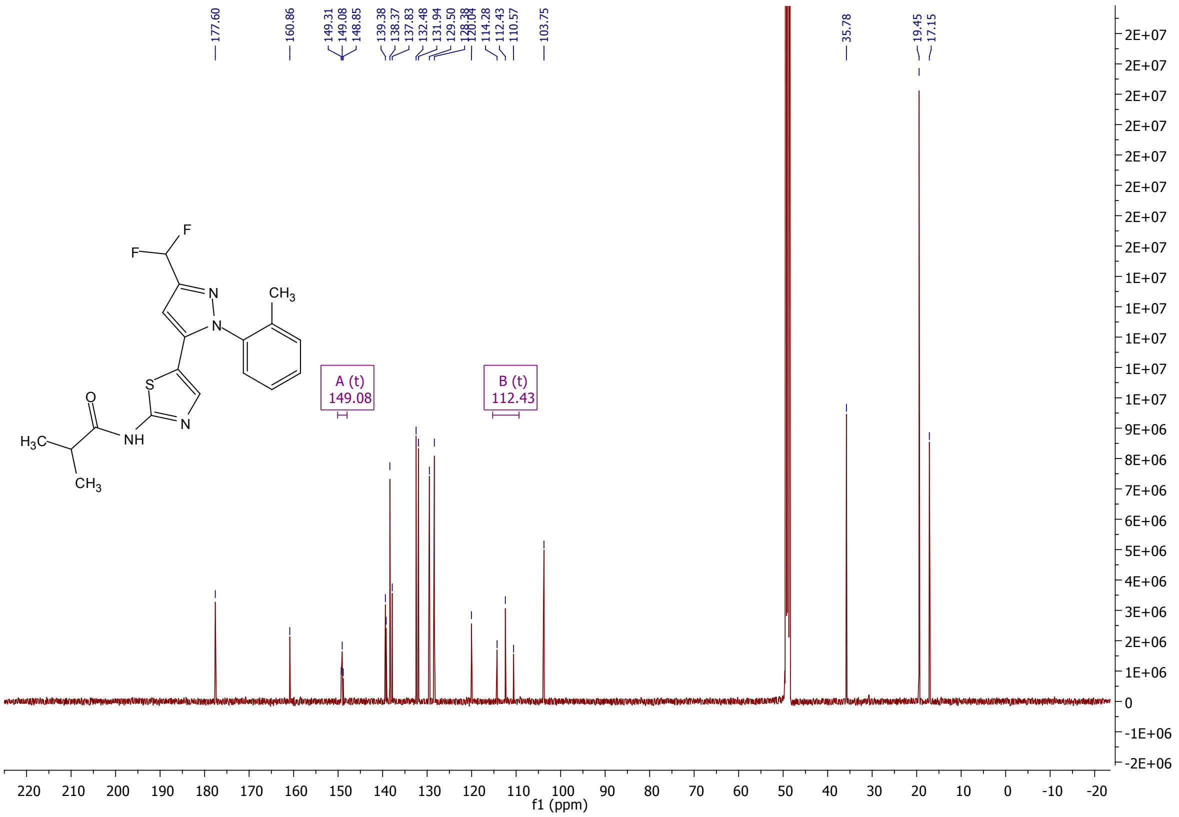

Figure S27. ^{13}C-NMR spectrum of compound 9i

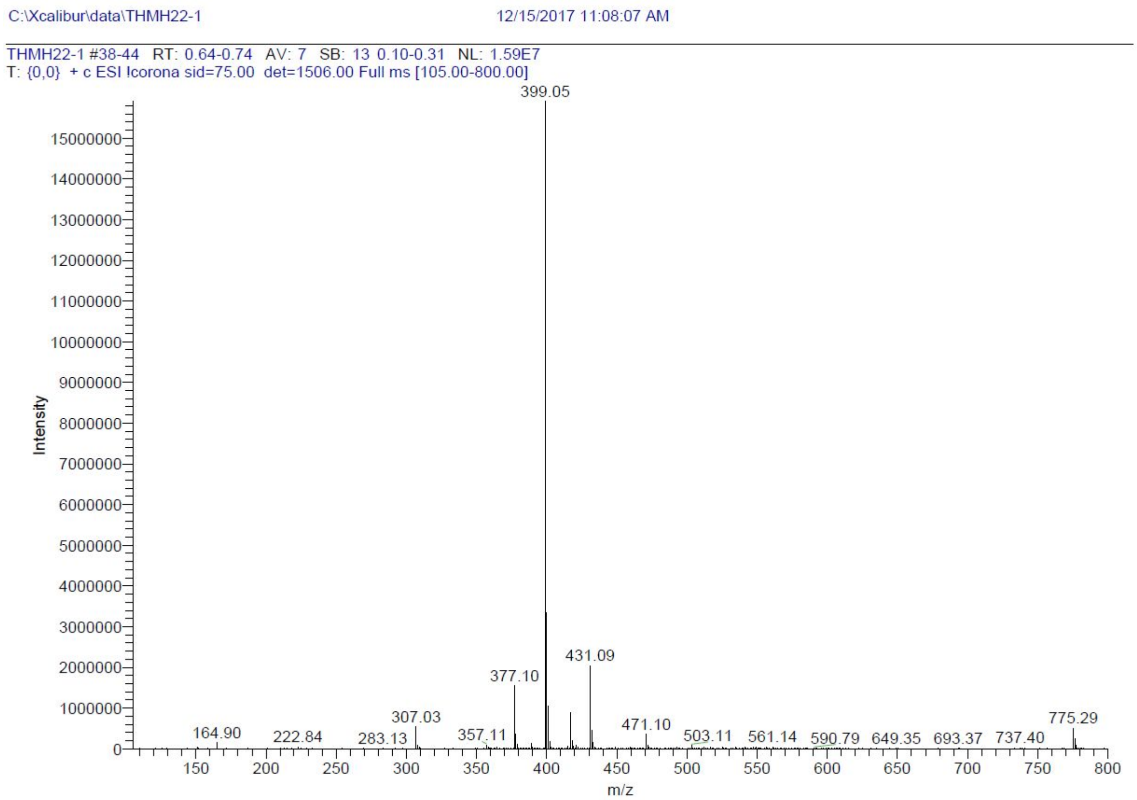

Figure S28. ESI-spectrum of compound 9i

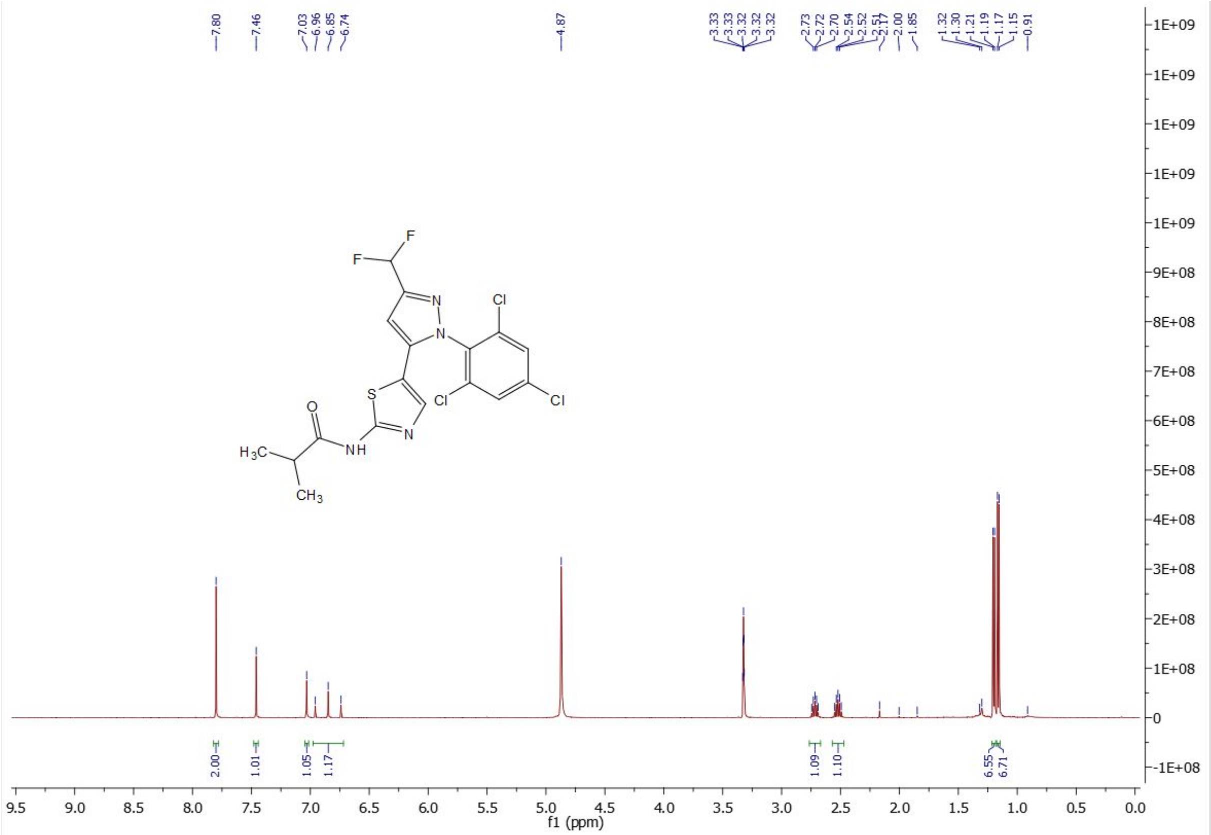

Figure S29. ^{1}H-NMR spectrum of compound 9j

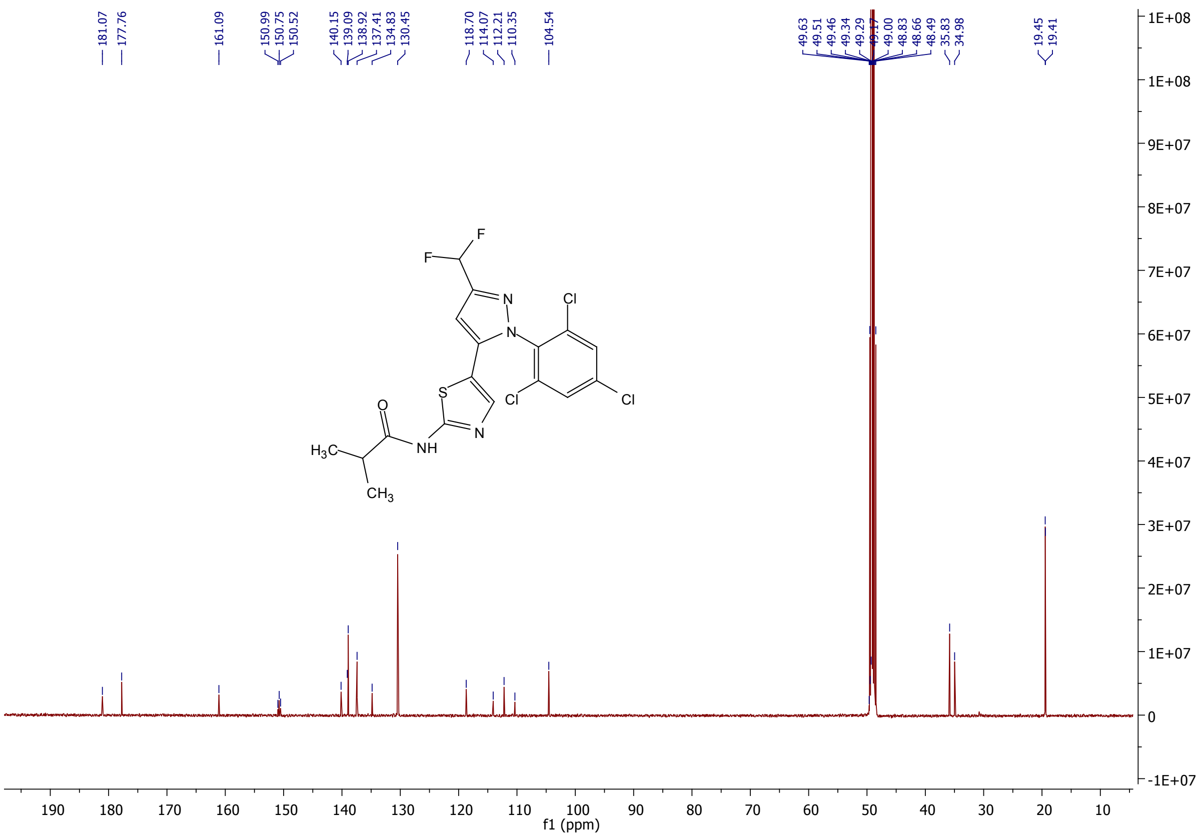

Figure S30. ^{13}C-NMR spectrum of compound 9j

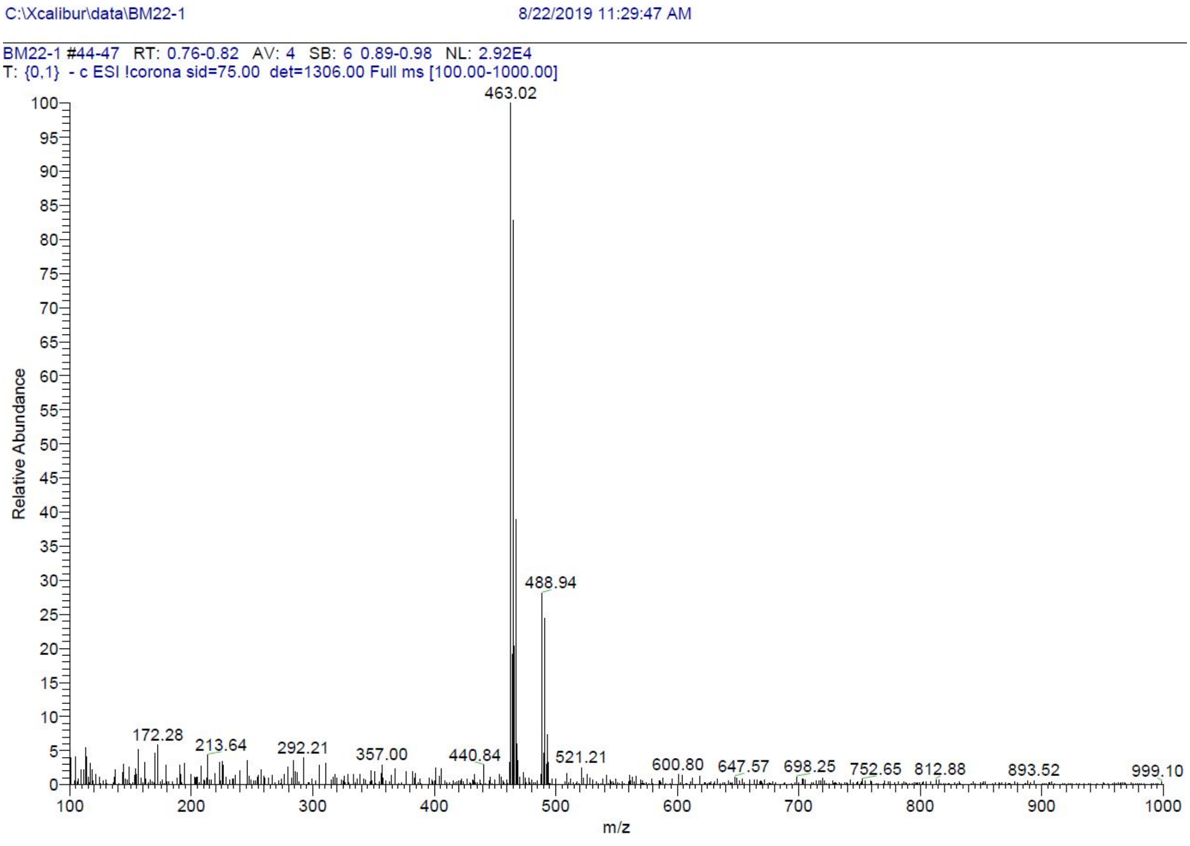

Figure S31. ESI spectrum of compound 9j

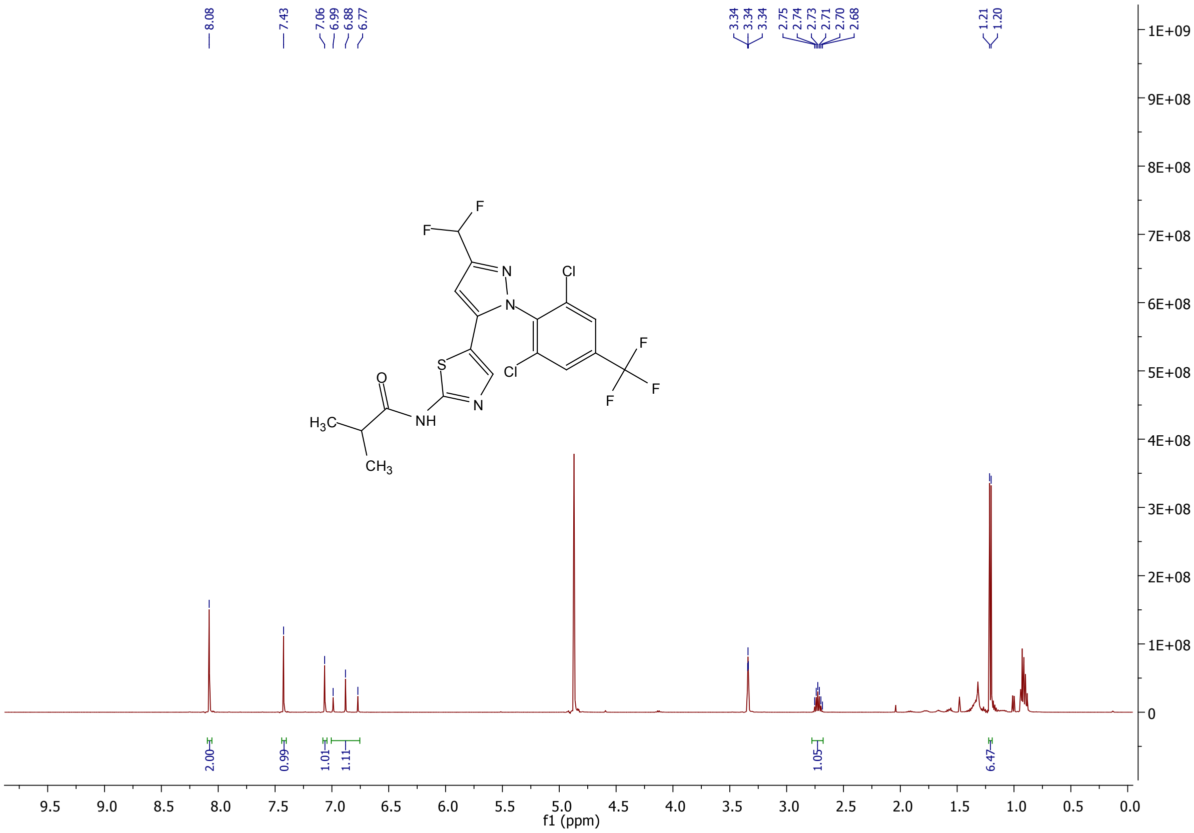

Figure S32. ^{1}H-NMR spectrum of compound 9k

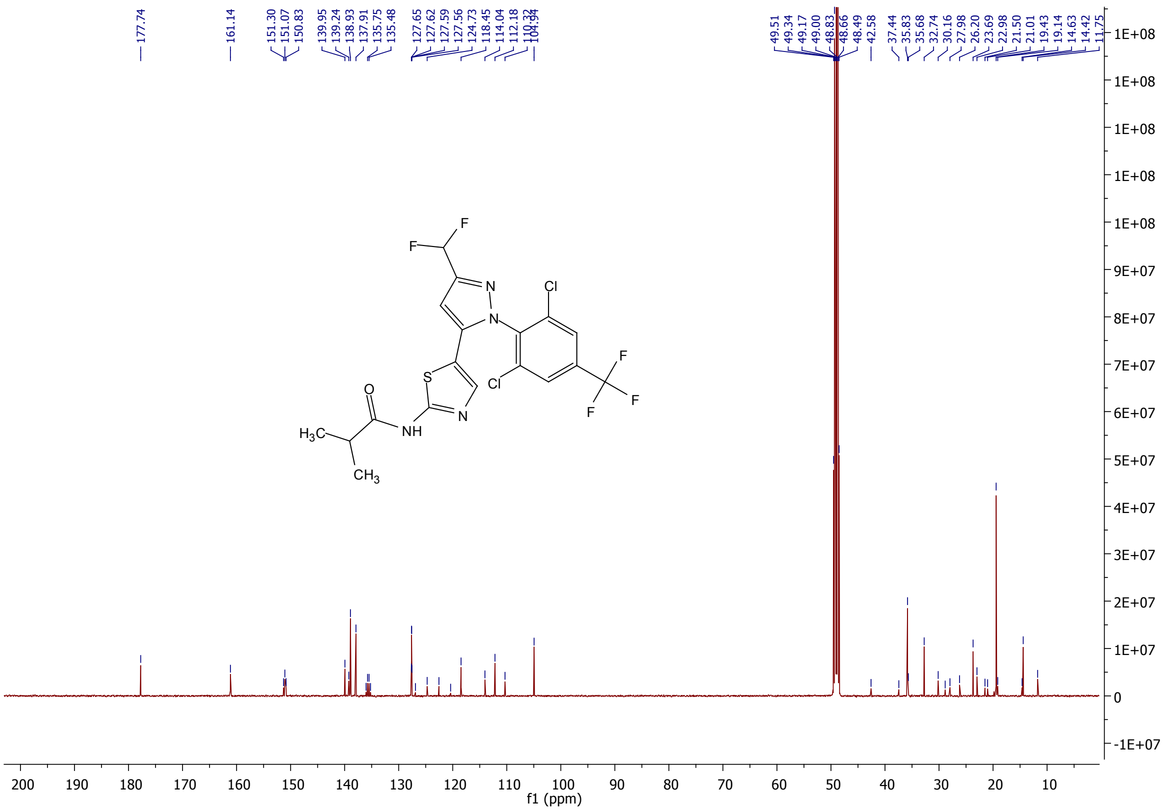

Figure S33. ^{13}C-NMR spectrum of compound 9k

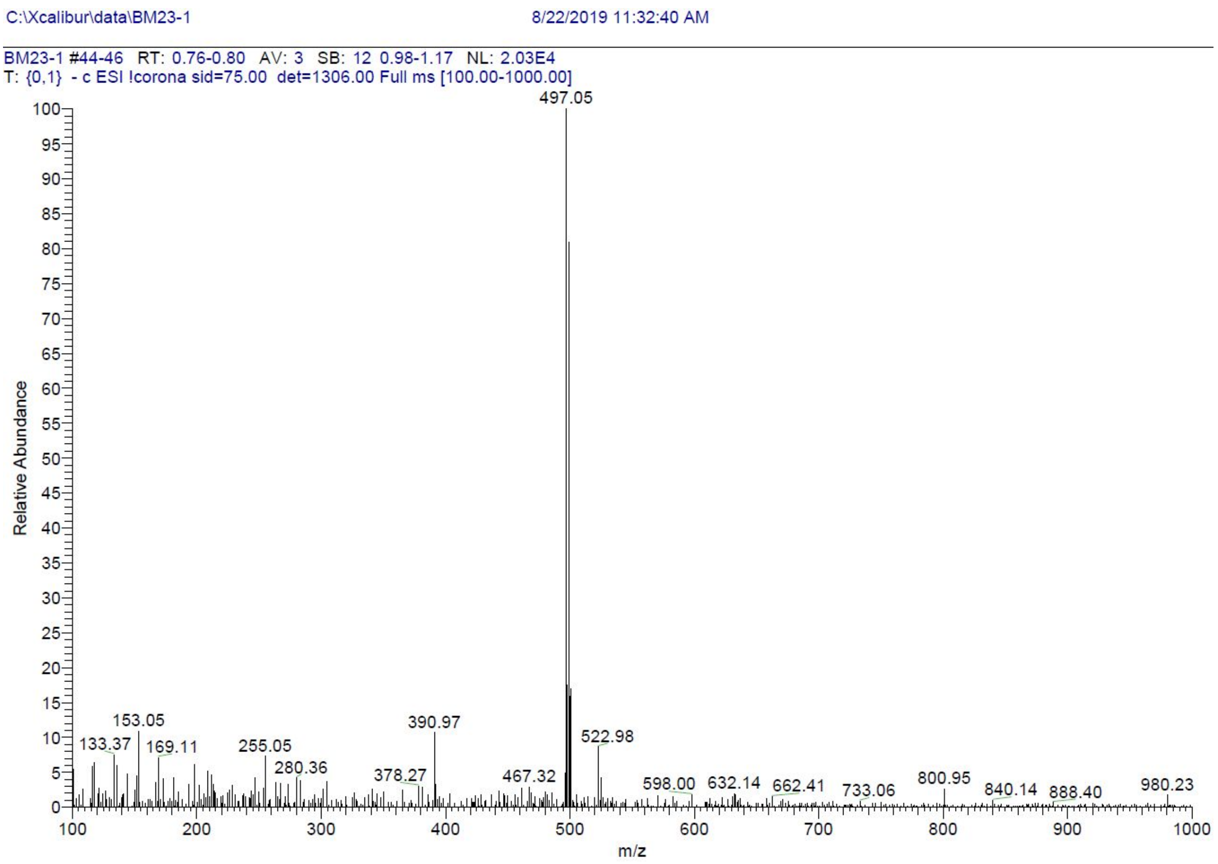

Figure S34. ESI spectrum of compound 9k

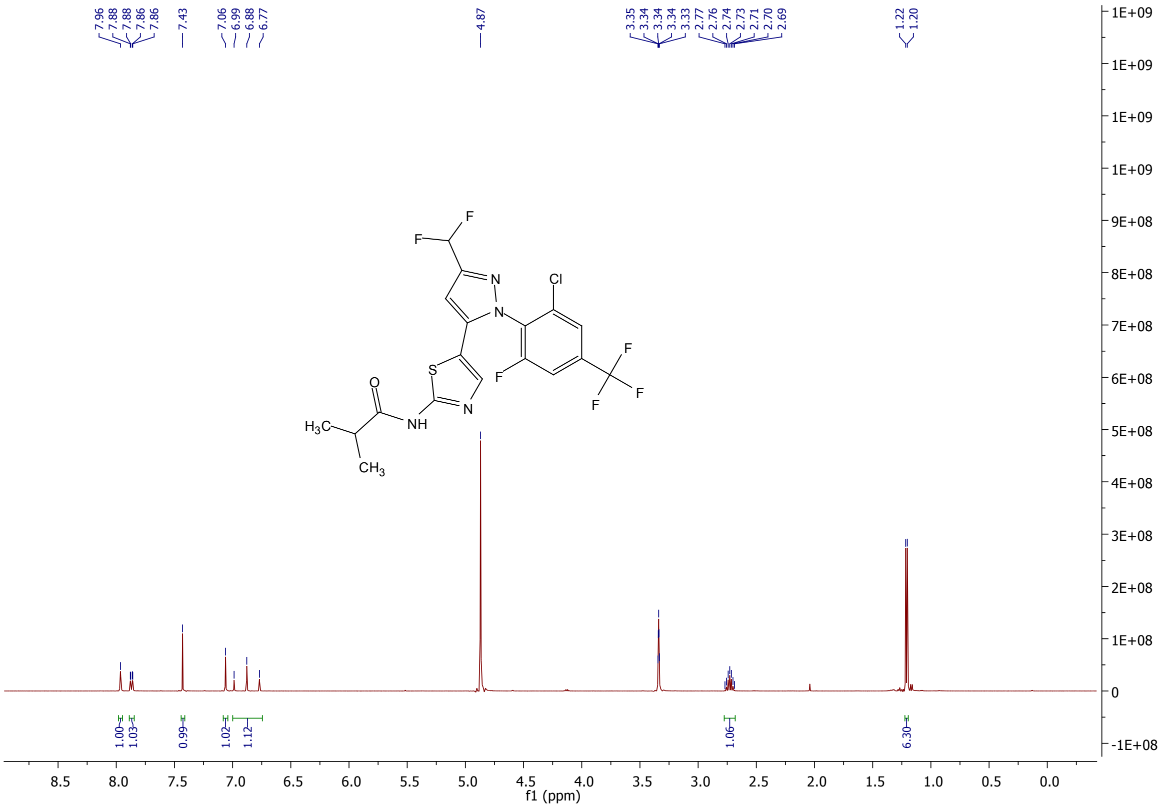

Figure S35. ^{1}H-NMR spectrum of compound 9l

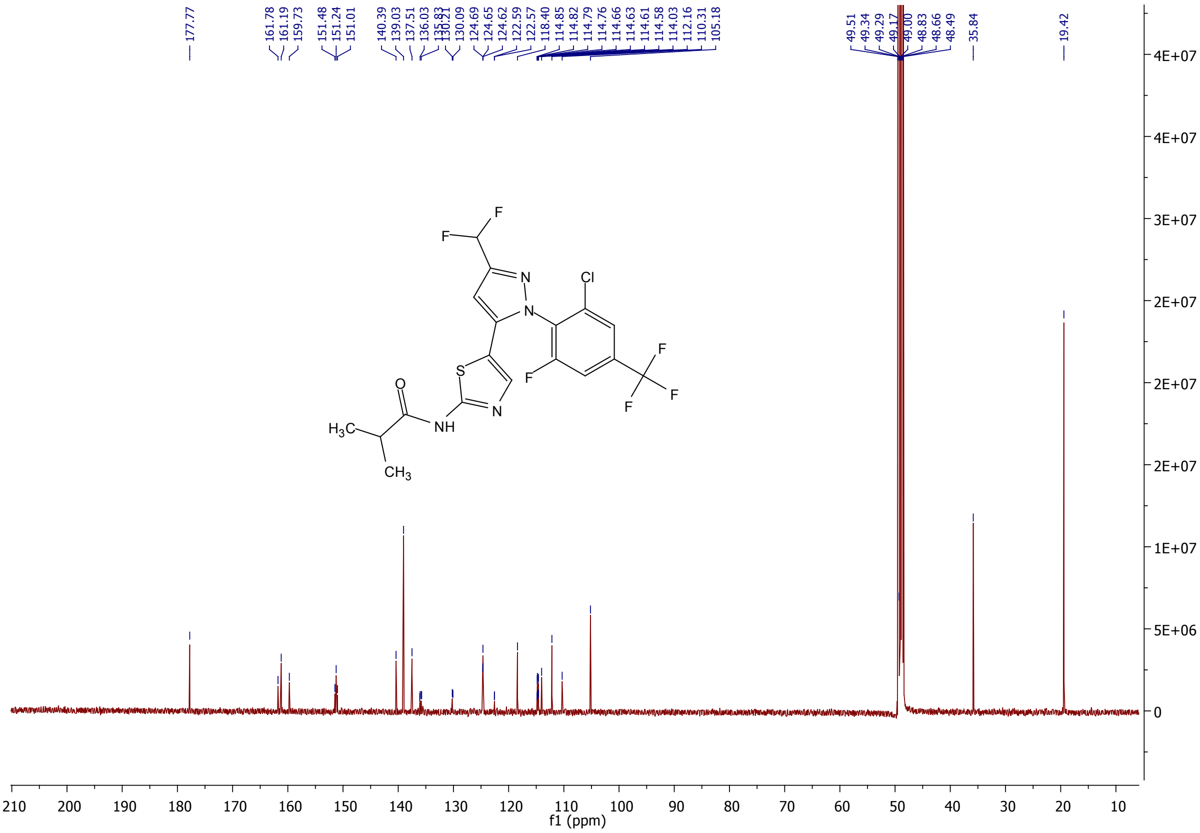

Figure S36. ^{13}C-NMR spectrum of compound 9l

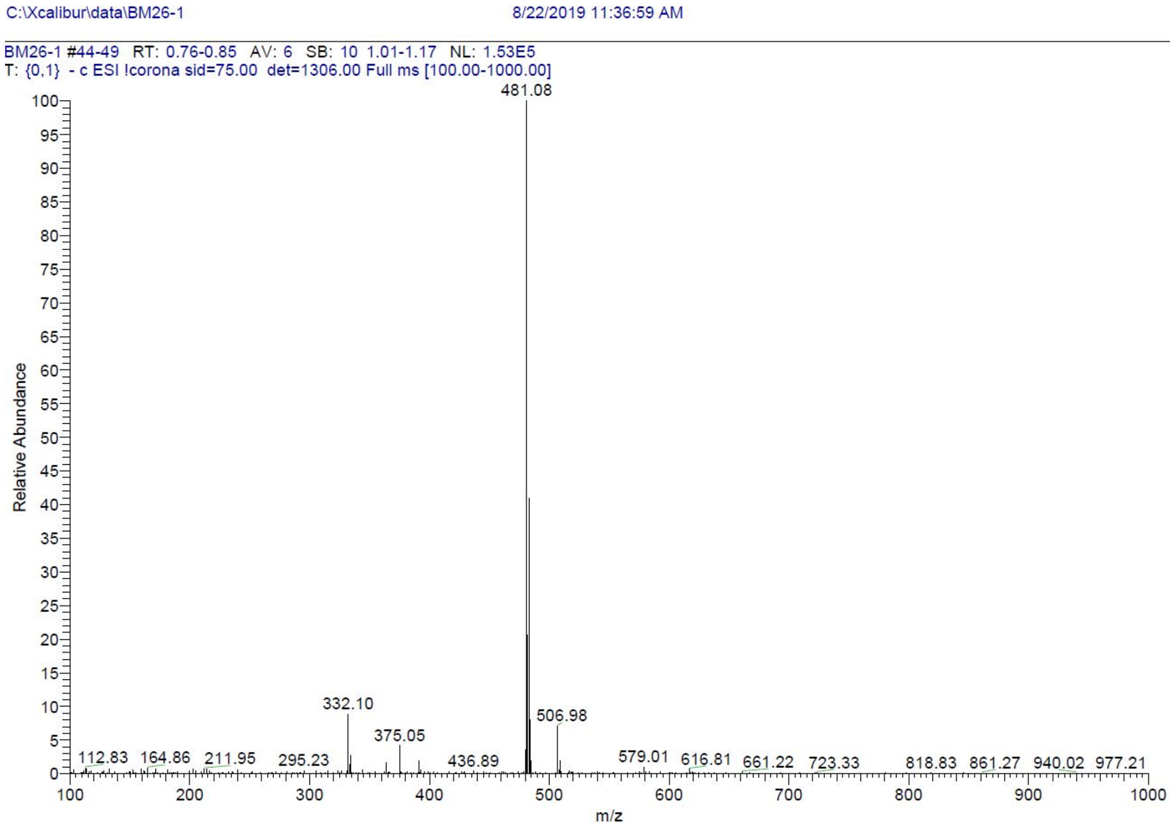

Figure S37. ESI spectrum of compound 9l

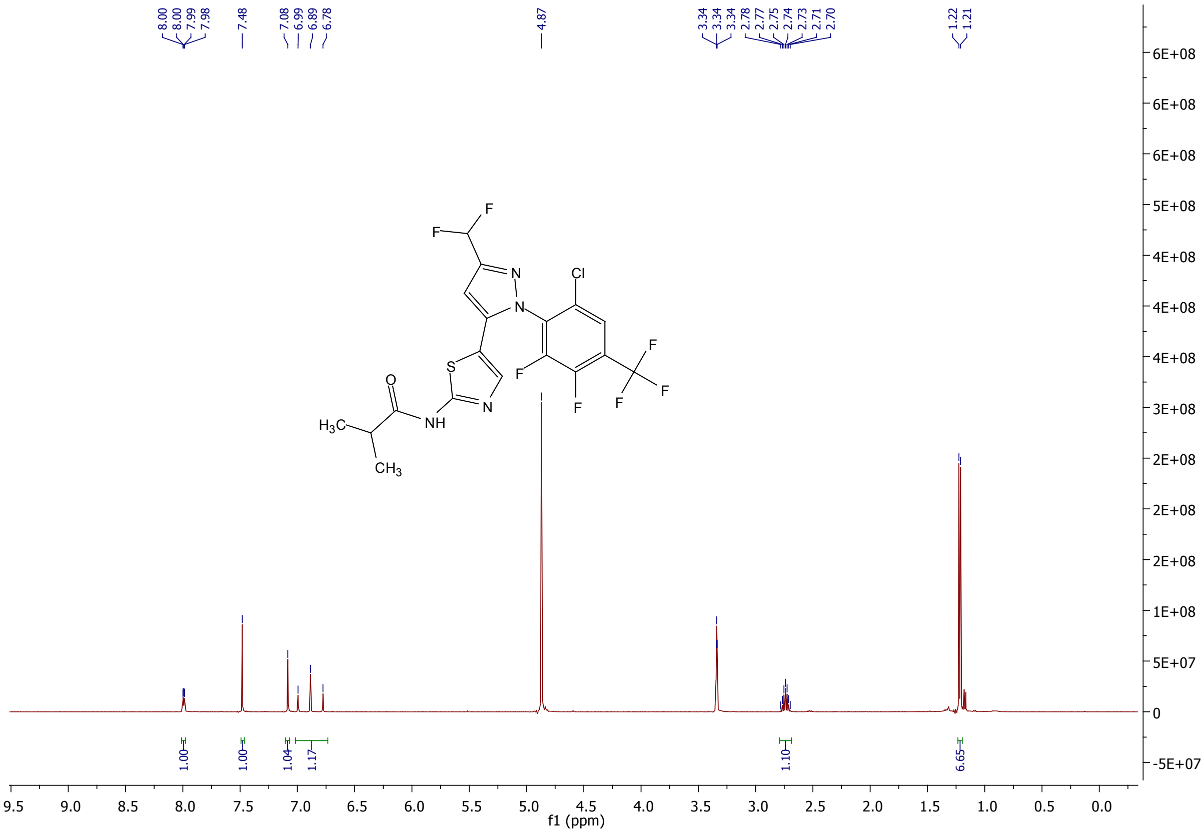

Figure S38. ^{1}H-NMR spectrum of compound 9m

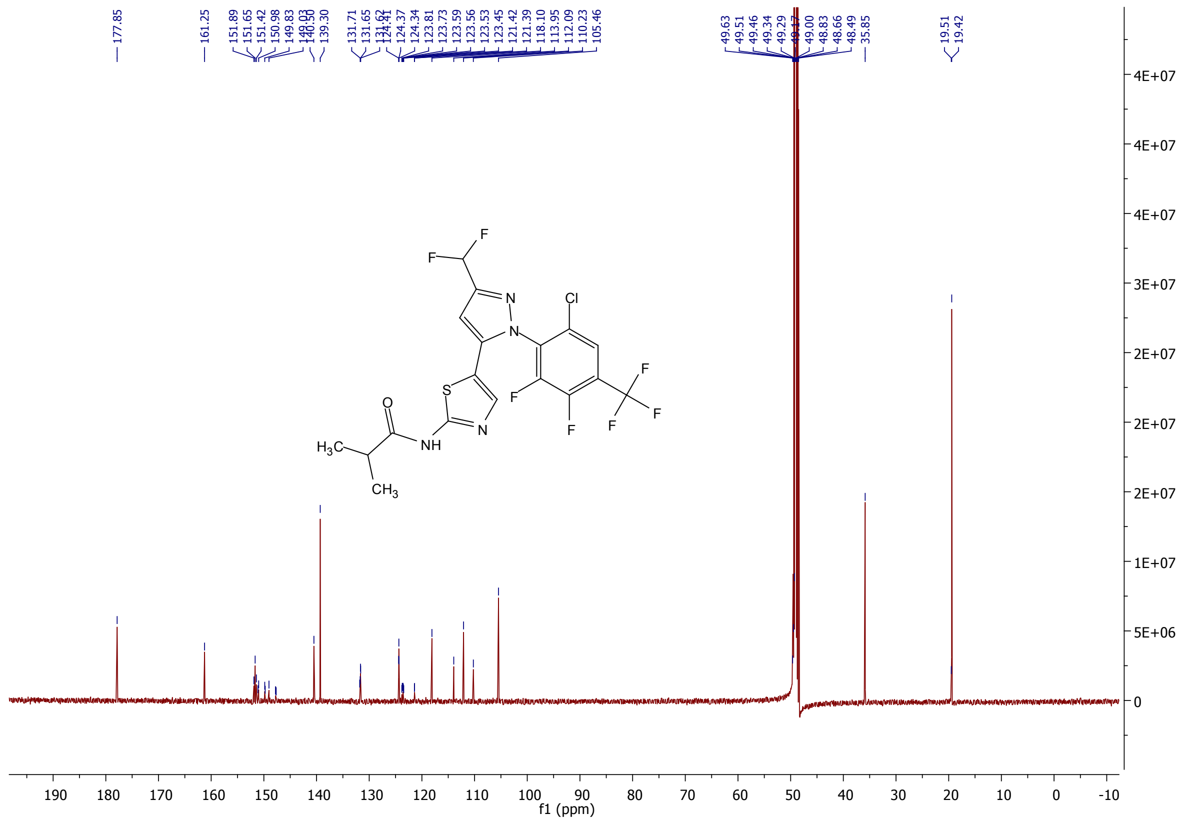

Figure S39. ^{13}C-NMR spectrum of compound 9m

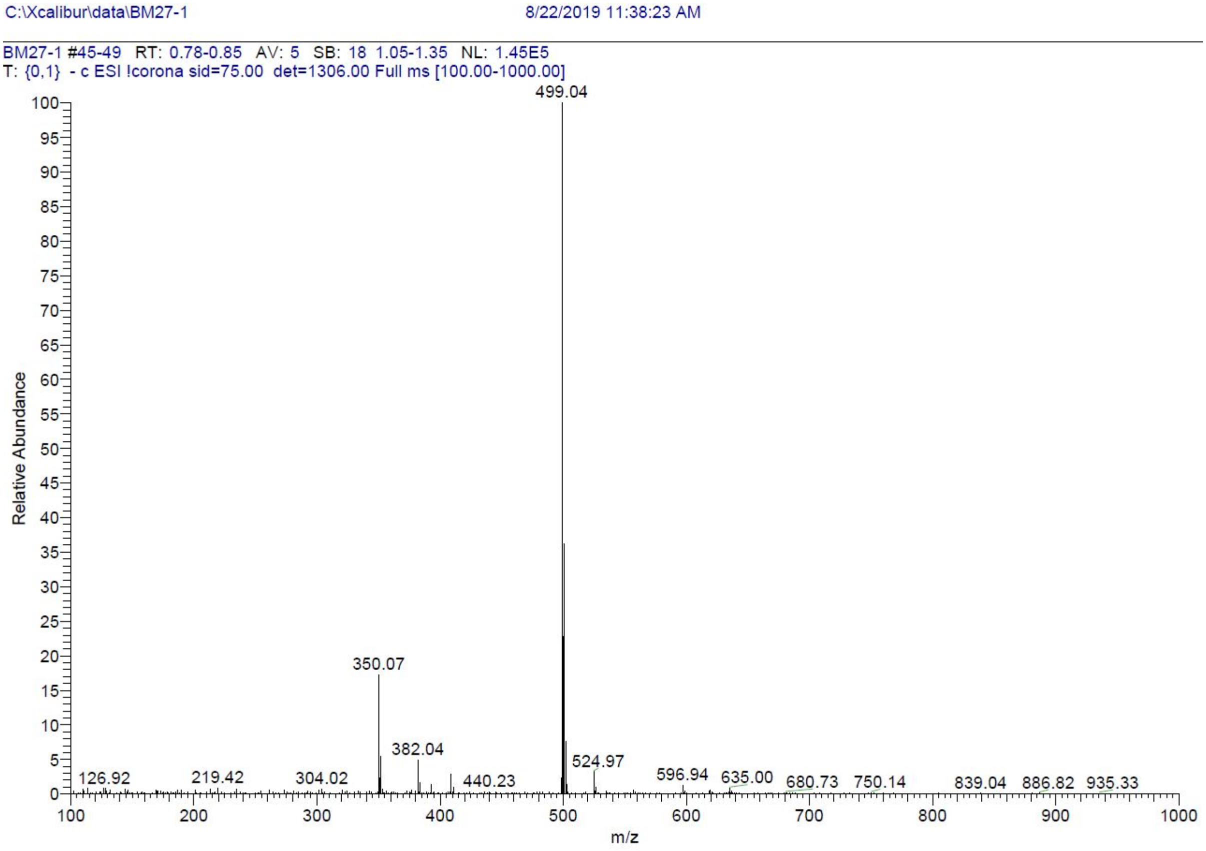

Figure S40. ESI spectrum of compound 9m

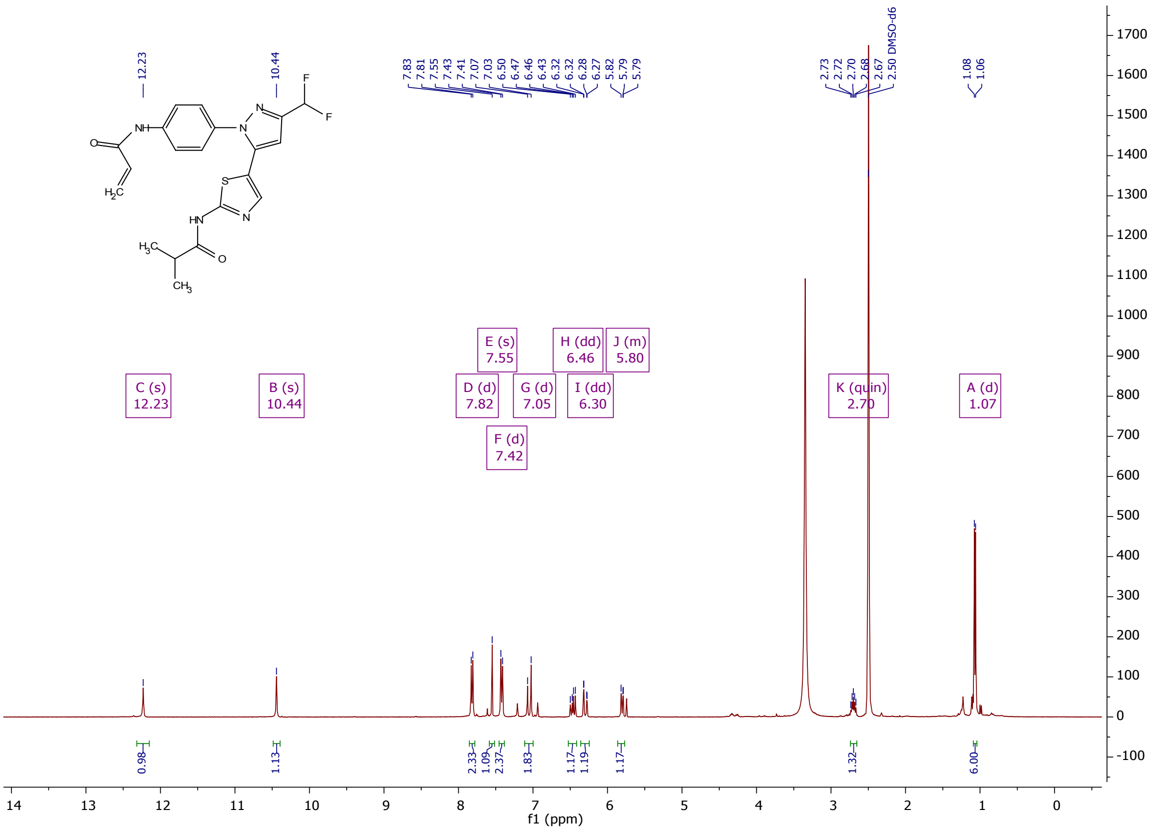

Figure S41. ^{1}H-NMR spectrum of compound 10

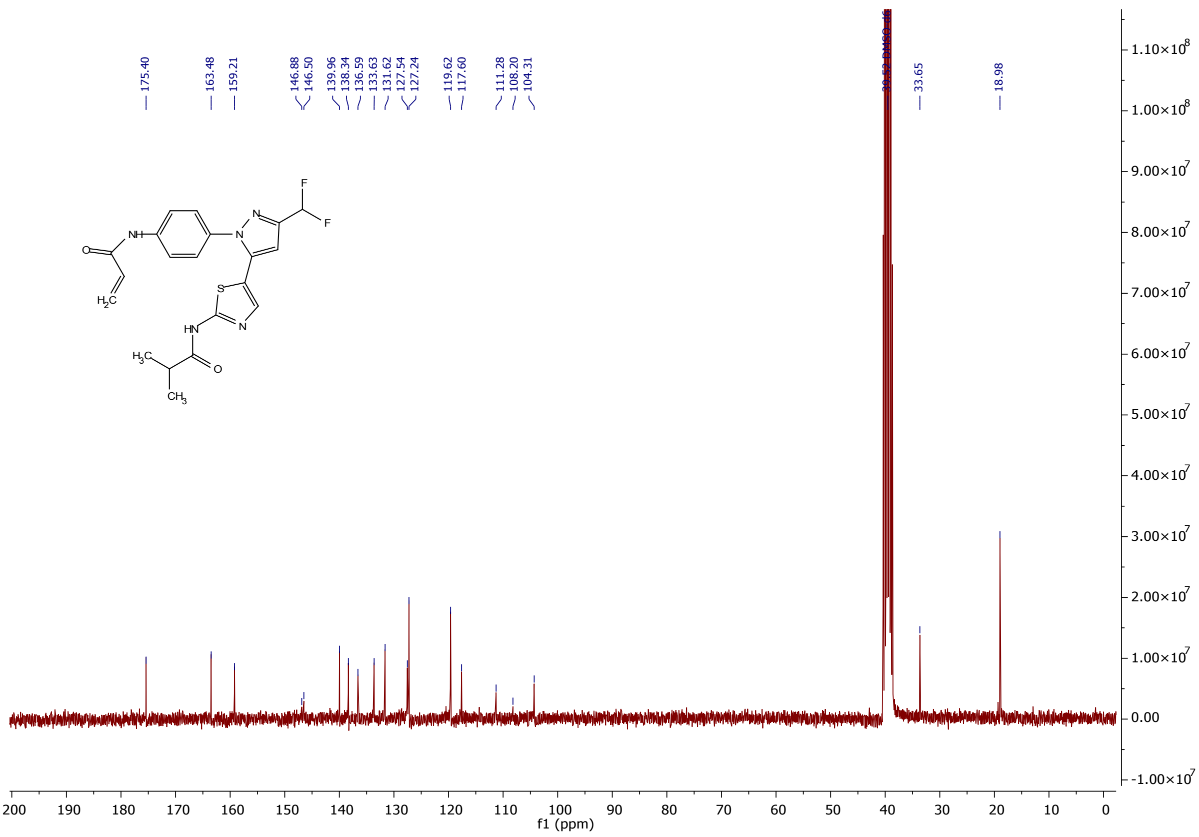

Figure S42. ^{1}H-NMR spectrum of compound 10

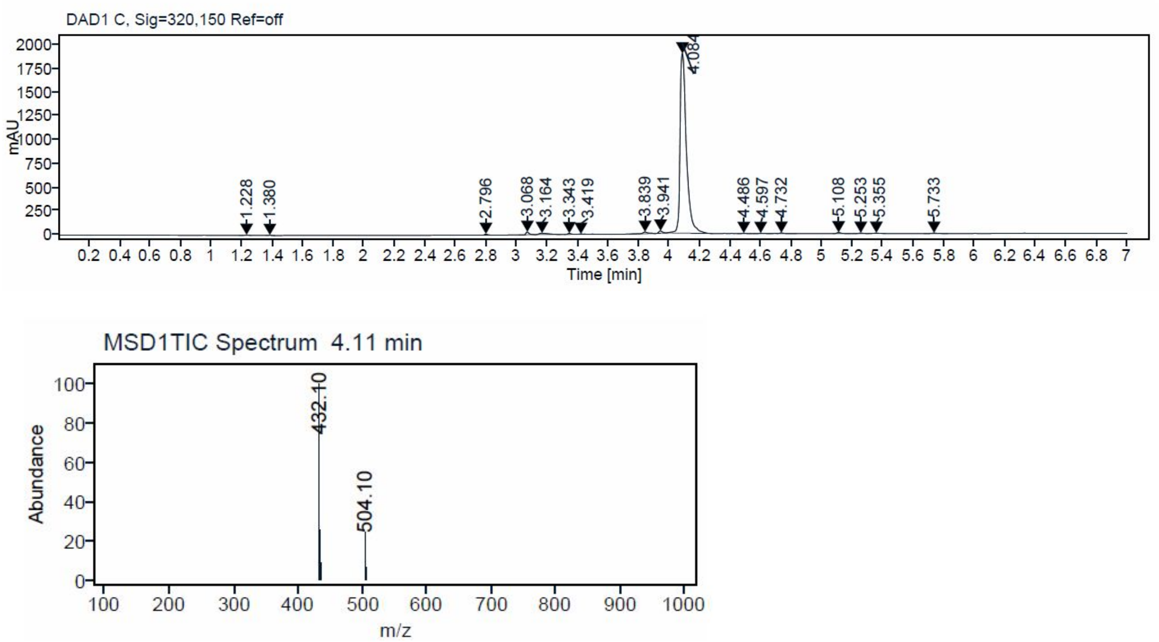

Figure S43. ESI-spectrum of compound 10

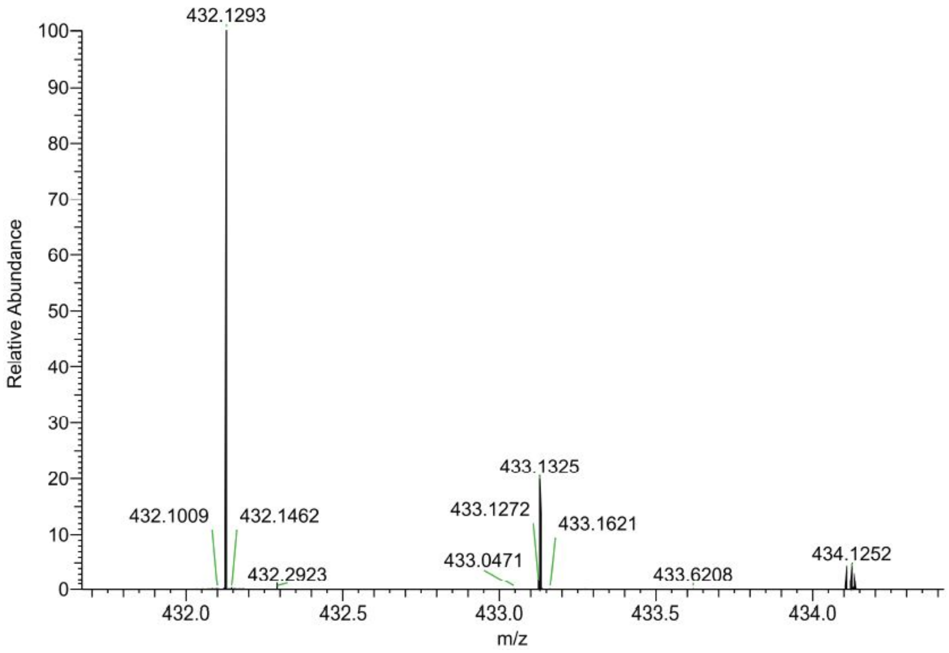

Figure S44. HRMS-spectrum of compound 10

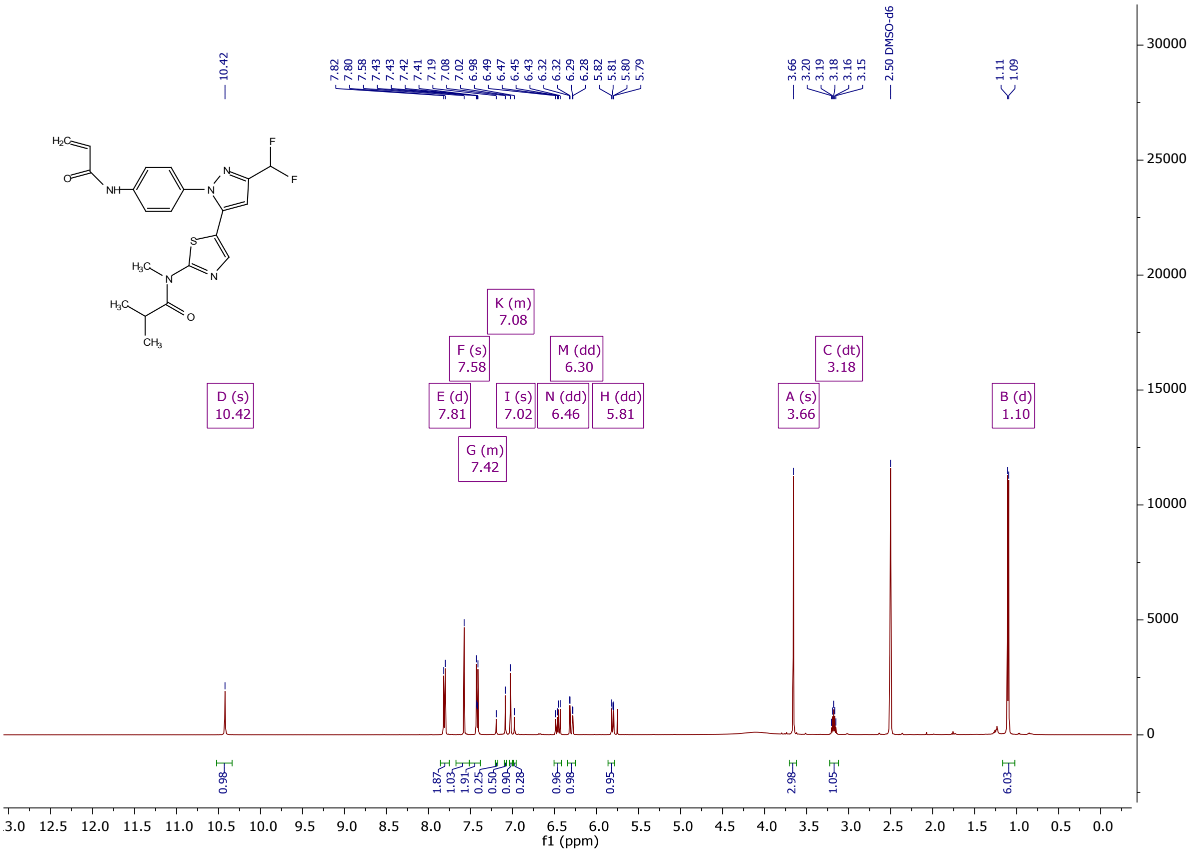

Figure S45. ^{1}H-NMR spectrum of compound 15

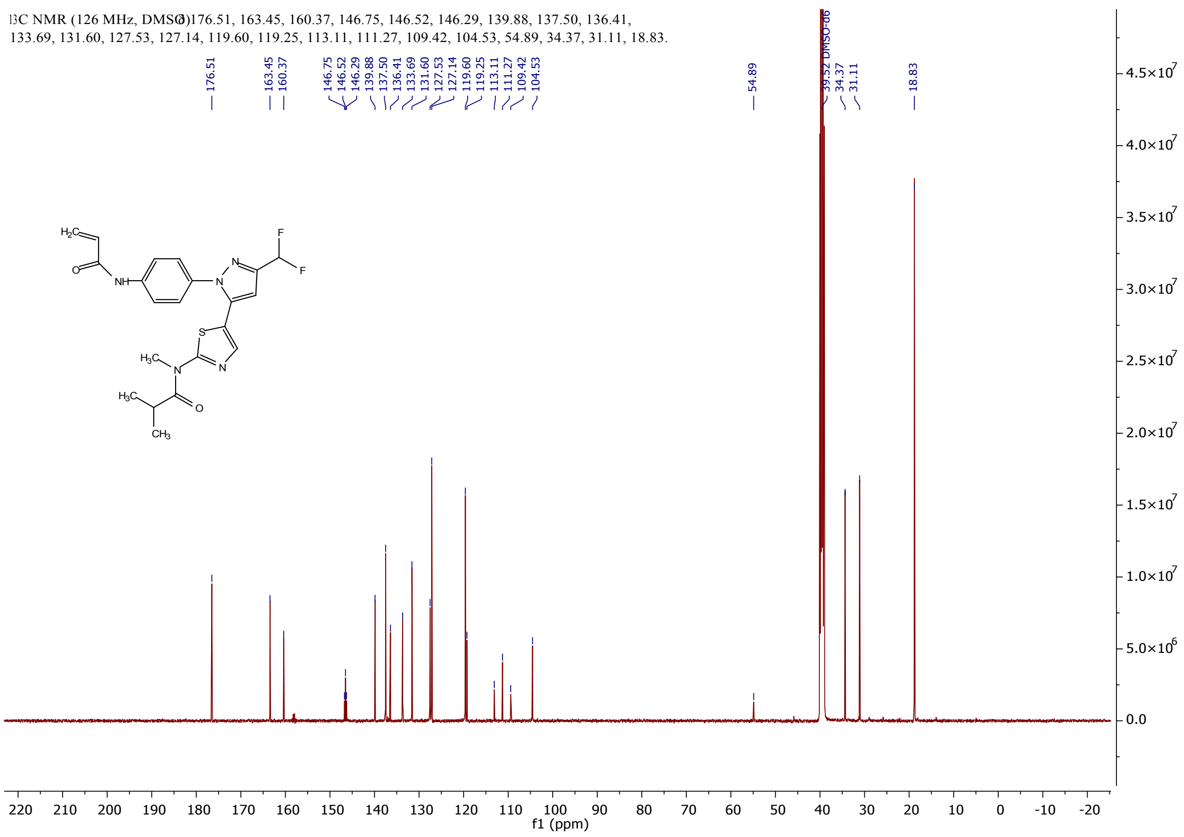

Figure S46. ^{13}C-NMR spectrum of compound 15

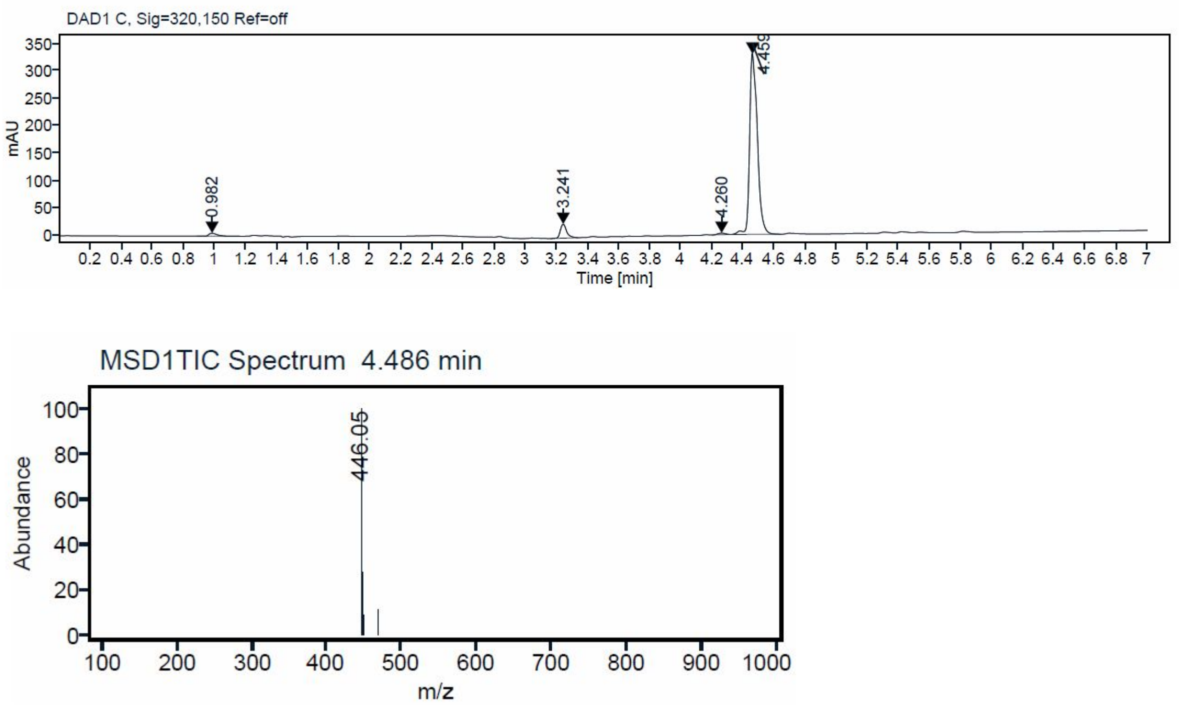

Figure S47. ESI-spectrum of compound 15

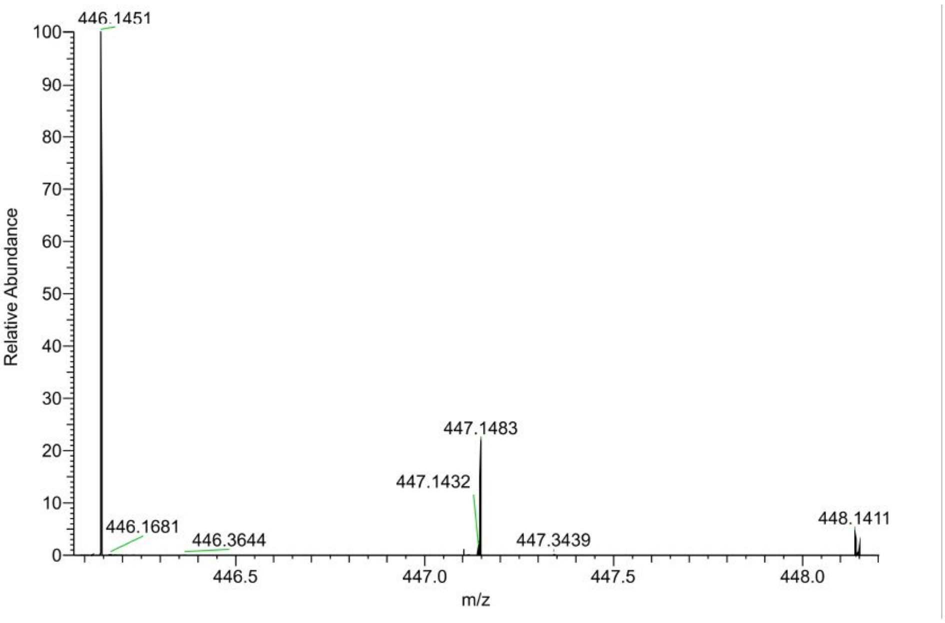

Figure S48. HRMS-spectrum of compound 15

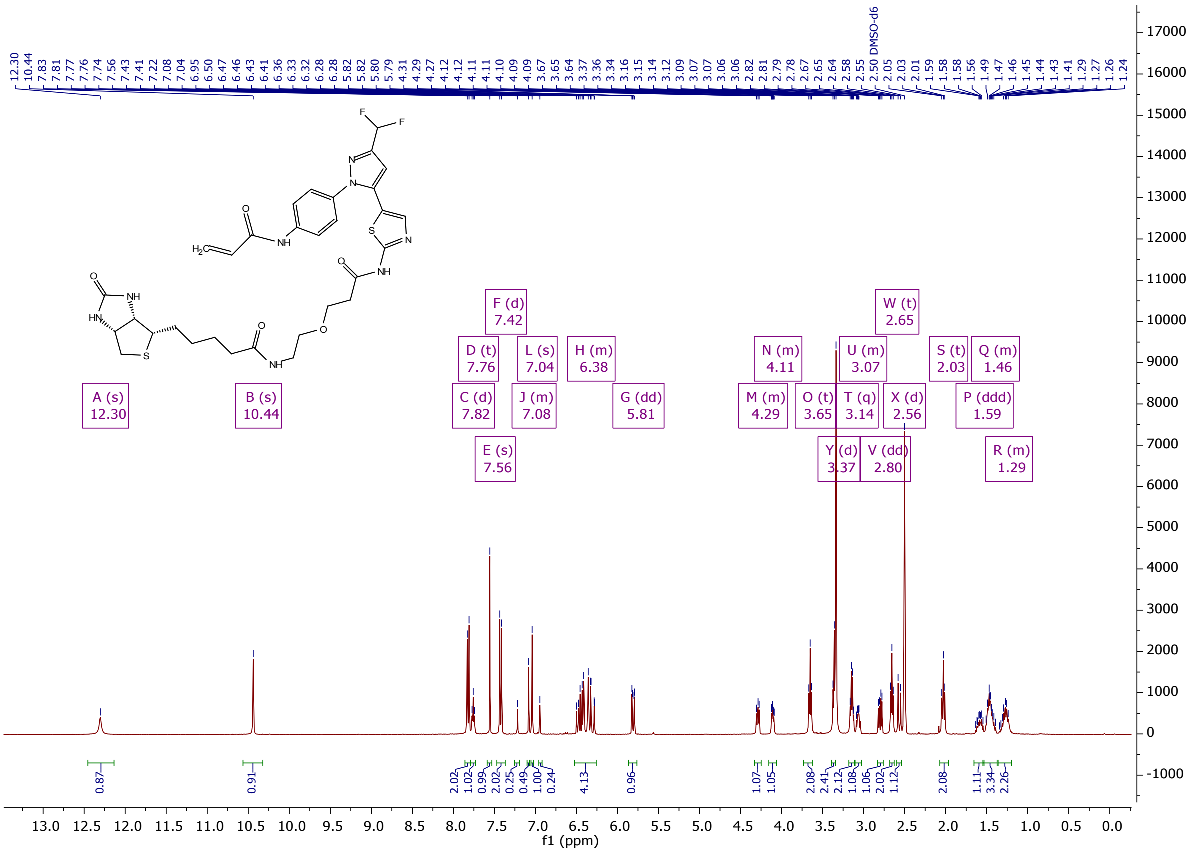

Figure S49. ^{1}H-NMR spectrum of compound 24

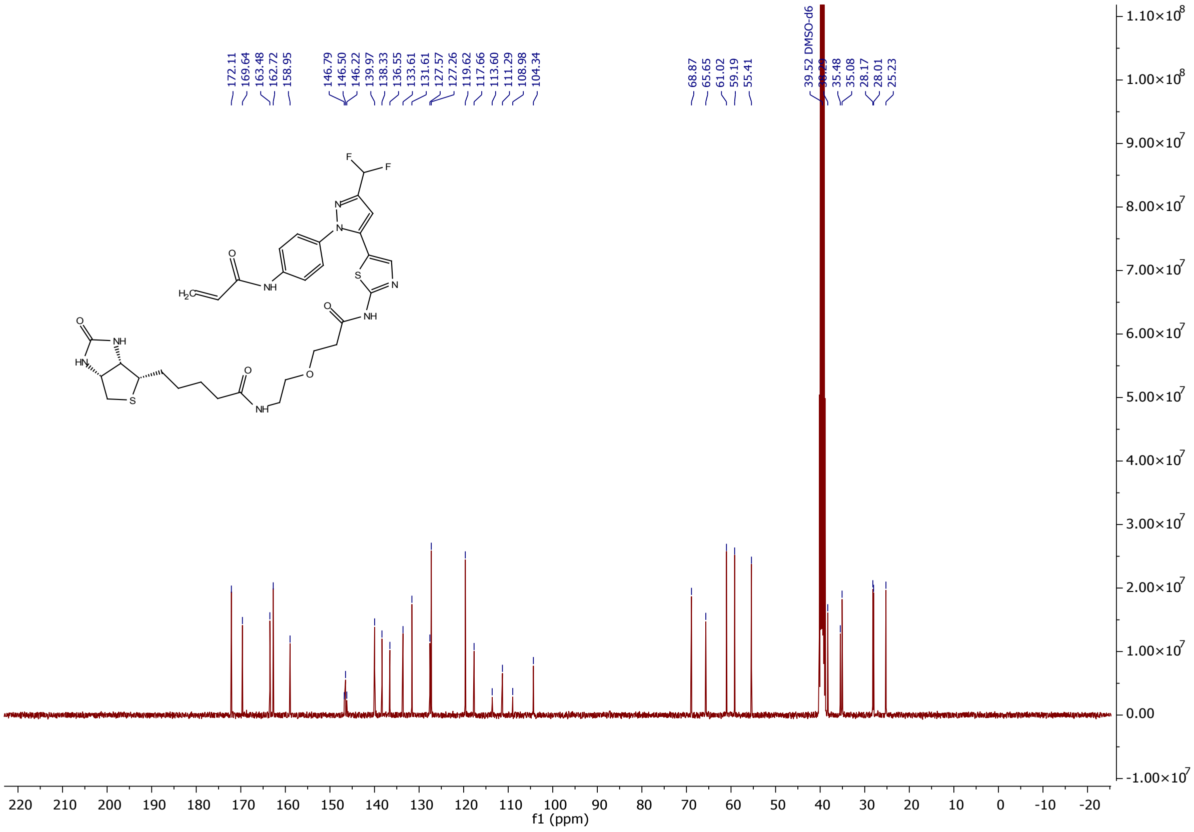

Figure S50. ^{13}C-NMR spectrum of compound 24

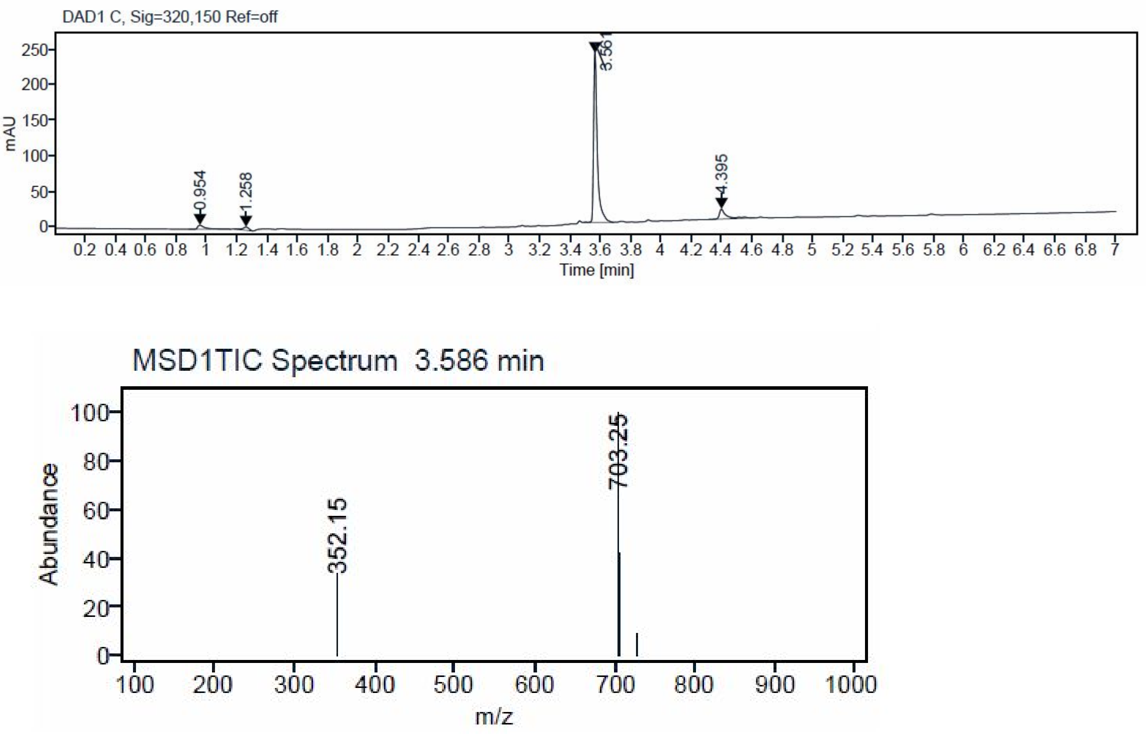

Figure S51. ESI-spectrum of compound 24

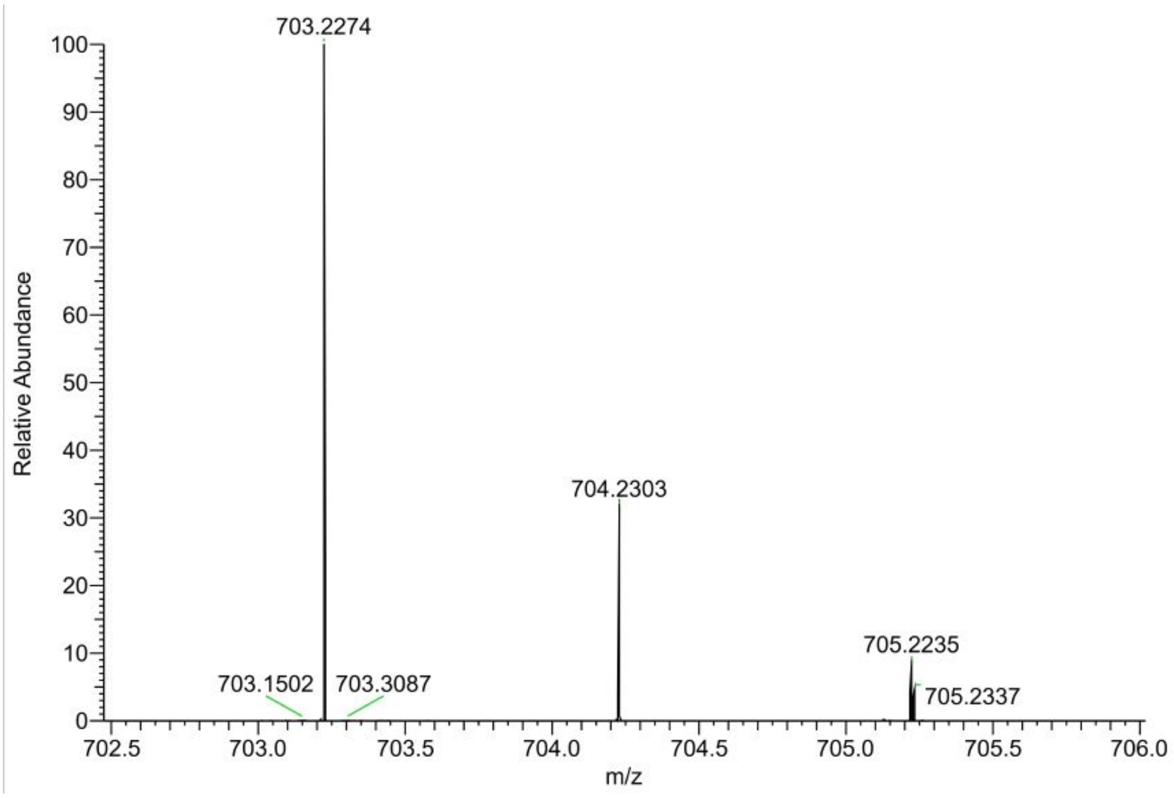

Figure S52. HRMS-spectrum of compound 24
